# RNALoc-LM: RNA subcellular localization prediction using pre-trained RNA language model

**DOI:** 10.1093/bioinformatics/btaf127

**Published:** 2025-03-22

**Authors:** Min Zeng, Xinyu Zhang, Yiming Li, Chengqian Lu, Rui Yin, Fei Guo, Min Li

**Affiliations:** School of Computer Science and Engineering, Central South University, Changsha, Hunan 410083, China; School of Computer Science and Engineering, Central South University, Changsha, Hunan 410083, China; School of Computer Science and Engineering, Central South University, Changsha, Hunan 410083, China; School of Computer Science, Key Laboratory of Intelligent Computing and Information Processing, Xiangtan University, Xiangtan, Hunan 411105, China; Department of Health Outcomes and Biomedical Informatics, University of Florida, Gainesville, FL 32603, United States; School of Computer Science and Engineering, Central South University, Changsha, Hunan 410083, China; School of Computer Science and Engineering, Central South University, Changsha, Hunan 410083, China

## Abstract

**Motivation:**

Accurately predicting RNA subcellular localization is crucial for understanding the cellular functions and regulatory mechanisms of RNAs. Although many computational methods have been developed to predict the subcellular localization of lncRNAs, miRNAs, and circRNAs, very few of them are designed to simultaneously predict the subcellular localization of multiple types of RNAs. In addition, the emergence of pre-trained RNA language model has shown remarkable performance in various bioinformatics tasks, such as structure prediction and functional annotation. Despite these advancements, there remains a significant gap in applying pre-trained RNA language models specifically for predicting RNA subcellular localization.

**Results:**

In this study, we proposed RNALoc-LM, the first interpretable deep-learning framework that leverages a pre-trained RNA language model for predicting RNA subcellular localization. RNALoc-LM uses a pre-trained RNA language model to encode RNA sequences, then captures local patterns and long-range dependencies through TextCNN and BiLSTM modules. A multi-head attention mechanism is used to focus on important regions within the RNA sequences. The results demonstrate that RNALoc-LM significantly outperforms both deep-learning baselines and existing state-of-the-art predictors. Additionally, motif analysis highlights RNALoc-LM’s potential for discovering important motifs, while an ablation study confirms the effectiveness of the RNA sequence embeddings generated by the pre-trained RNA language model.

**Availability and implementation:**

The RNALoc-LM web server is available at http://csuligroup.com:8000/RNALoc-LM. The source code can be obtained from https://github.com/CSUBioGroup/RNALoc-LM.

## 1 Introduction

RNA subcellular localization refers to the process by which RNA molecules are distributed and directed to specific locations within a cell. This process is crucial for cellular function and the regulation of gene expression. Long non-coding RNAs (lncRNAs), circular RNAs (circRNAs), and microRNAs (miRNAs) are three important classes of RNA molecules, each playing distinct roles in cellular processes. They are involved in the regulation of gene expression and are associated with various diseases (Rackham and Brown 2004, Zeng *et al.* 2020, Lu *et al.* 2020, 2021). Accurate prediction of their subcellular localization is essential for understanding their functions and interactions within the cell (Rinn and Chang 2012, Xuan *et al.* 2016, Lu *et al.* 2020, 2021).

Traditionally, single-molecule fluorescence in situ hybridization (smFISH) is regarded as the gold standard for RNA subcellular localization. While the high-resolution imaging data provided by smFISH are ideal for precisely localizing RNA within cells, the technique is costly, time-consuming, and technically demanding (Femino *et al.* 1998). Given these constraints, there is significant value in developing accurate and reliable computational methods to predict RNA subcellular localization.

Currently, many machine learning and deep-learning-based methods have been proposed for predicting RNA subcellular localization, directly from RNA sequences (Garg *et al.* 2020, Wang *et al.* 2021, Wang *et al.* 2023). To the best of our knowledge, lncLocator (Cao *et al.* 2018) is the first predictor developed for predicting the subcellular localization of lncRNA. This was followed by several other methods, including iLoc-lncRNA (Su *et al.* 2018), DeepLncRNA (Gudenas and Wang 2018), Locate-R (Ahmad *et al.* 2020), LncLocation(Feng *et al.* 2020), iLoc-lncRNA (2.0) (Zhang *et al.* 2022a,b), DeepLncLoc (Zeng *et al.* 2022), GraphLncLoc (Li *et al.* 2023), SGCL-LncLoc (Li *et al.* 2024), and LncLocFormer (Zeng *et al.* 2023) all aiming at predicting lncRNA subcellular localization. For miRNA, a type of small, non-coding RNA molecule, several computational methods have been developed for subcellular localization identification, including miRLocator (Xiao *et al.* 2018), miRNALoc (Meher *et al.* 2020), MirLocPredictor(Asim *et al.* 2020), L2S-MirLoc (Asim *et al.* 2021), and iLoc-miRNA (Zhang *et al.* 2022a,b), using miRNA sequence information. Additionally, MiRGOFS (Yang *et al.* 2018) uses Gene Ontology (GO) semantic similarity measurements, while MiRLoc (Xu *et al.* 2022) and DAmiRLocGNet (Bai *et al.* 2023) leverage miRNA functional similarities. For circRNA, Circ-LocNet (Asim *et al.* 2022) is the first computational method focused on predicting the subcellular localization of circRNAs. CellCircLoc (Zeng *et al.* 2025) further enhances predictions by accommodating circRNA subcellular localization across different cell lines.

Although existing computational methods have made significant contributions to the prediction of RNA subcellular localization, only RNAlight (Yuan *et al.* 2023) and DeepLocRNA (Wang *et al.* 2024) have been designed for predict the subcellular localization of multiple types of RNAs. However, the two methods have certain limitations. In more detail, they mainly rely traditional encoding techniques for processing RNA sequences, such as k-mer (Bessière *et al.* 2024) and one-hot encoding (Zou *et al.* 2019), which overlook the rich biological information that is inherent in RNA sequences. Recent advancement in pre-trained RNA language models have revolutionized the field of RNA bioinformatics(Ding *et al.* 2022), significantly enhancing downstream tasks such as RNA structure prediction. However, there remains a notable gap in the application of pre-trained RNA language models specifically for predicting RNA subcellular localization. These RNA language models are trained on hundreds of millions of natural RNA sequences, capturing multi-dimension biological information that encompasses structural and functional properties as well as evolutionary information. This provides a rich representation that could greatly improve predictions of RNA subcellular localization.

In this study, we introduced RNALoc-LM, a groundbreaking and interpretable deep-learning framework that uses a pre-trained RNA language model to forecast the subcellular localization of RNA molecules. RNALoc-LM utilizes a pre-trained RNA language model, RNA-FM, to encode RNA sequences, generating embedding representations that are processed through a TextCNN module to extract local features. A BiLSTM module is then used to capture long-range dependencies and contextual information. Additionally, RNALoc-LM uses a multi-head attention mechanism to focus on the important segments of the RNA sequence. Finally, a fully connected layer is used to perform the RNA subcellular localization prediction task.

To evaluate the performance of RNALoc-LM, we compared it with deep-learning baseline models and existing state-of-the-art predictors. The results demonstrate that RNALoc-LM achieves superior performance in predicting the subcellular localization of three types of RNAs: lncRNA, circRNA, and miRNA, consistently outperforming all the benchmarks. Furthermore, motif analysis reveals that RNALoc-LM captures important sequence motifs, enhancing the interpretability of the model’s predictions. An ablation study further confirms the effectiveness of the RNA sequence embeddings encoded by the RNA-FM model. Finally, to facilitate the use of RNALoc-LM, we developed a user-friendly web server.

## 2 Materials and methods

### 2.1 Benchmark dataset

Building a high-quality benchmark dataset is a fundamental prerequisite for the study. The RNALocate database (Zhang *et al.* 2017) is a comprehensive database dedicated to RNA subcellular localization, with significant updates from its first version (v1.0) to the second version (v2.0). The Cancer-Specific CircRNA Database (CSCD) (Feng *et al.* 2022) specifically focuses on circular RNAs (circRNAs) associated with various cancers. We collected lncRNA and miRNA subcellular localization data from RNALocate v1.0 (Zhang *et al.* 2017) and RNALocate v2.0 (Cui *et al.* 2022) databases, as well as circRNA subcellular localization data from CSCD 2.0 database (Feng *et al.* 2022). The benchmark dataset was constructed through the following steps:

1. We collected 11 520 lncRNA-associated subcellular localization entries from RNALocate v1.0 and RNALocate v2.0 databases. Additionally, we collected 7449 miRNA-associated subcellular localization entries from the two datasets. Also, we retrieved 1 013 461 circRNA-associated subcellular localization entries from the CSCD 2.0 database.
2. Some lncRNAs, miRNAs, and circRNAs have multiple subcellular localization entries in these databases, hence, we merged data sharing the same gene symbols and removed entries lacking sequence information.
3. We focused on RNAs that were localized to a single subcellular compartment in our study, since most lncRNAs, miRNAs, and circRNAs are associated with a single subcellular localization in these databases.
4. Considering the balance of the collected dataset, we excluded subcellular localization categories with very few samples. In addition, due to the ambiguous annotations of cytoplasm and cytosol in early literature, we only considered the data of cytoplasmic localization (Wang *et al.* 2021). Specifically, for lncRNAs, only data of cytoplasm and nucleus were retained. For miRNAs, we aligned with the iLoc-miRNA, categorizing miRNA localization into extracellular regions (exosomes, microvesicles, extracellular vesicles, or plasma membrane vesicles) and intracellular regions (chromatin, cytoplasm, plasma membrane, endoplasmic reticulum membrane, nucleolus, mitochondria or nucleus, processing body) based on their subcellular localization. For circRNAs, we only focused on the nucleus and cytoplasm categories.
5. Then, we used the CD-HIT-EST (Huang *et al.* 2010) tool to remove redundant sequences from the three RNA datasets with a cutoff value of 80%.

Finally, we obtained a benchmark dataset containing 1003 lncRNAs, 1124 miRNAs, and 27 682 circRNAs. Table 1 shows the subcellular localization distribution of different RNA types in the benchmark dataset.

**Table 1.** Distribution of subcellular localization data for different RNA types in the benchmark dataset.

RNA type	Subcellular localization	No. of samples
lncRNA	Nucleus	636
	Cytoplasm	367
miRNA	Extracellular region	750
	Intracellular region	374
circRNA	Nucleus	11 660
	Cytoplasm	16 022

### 2.2 RNALoc-LM architecture

**Figure 1.**
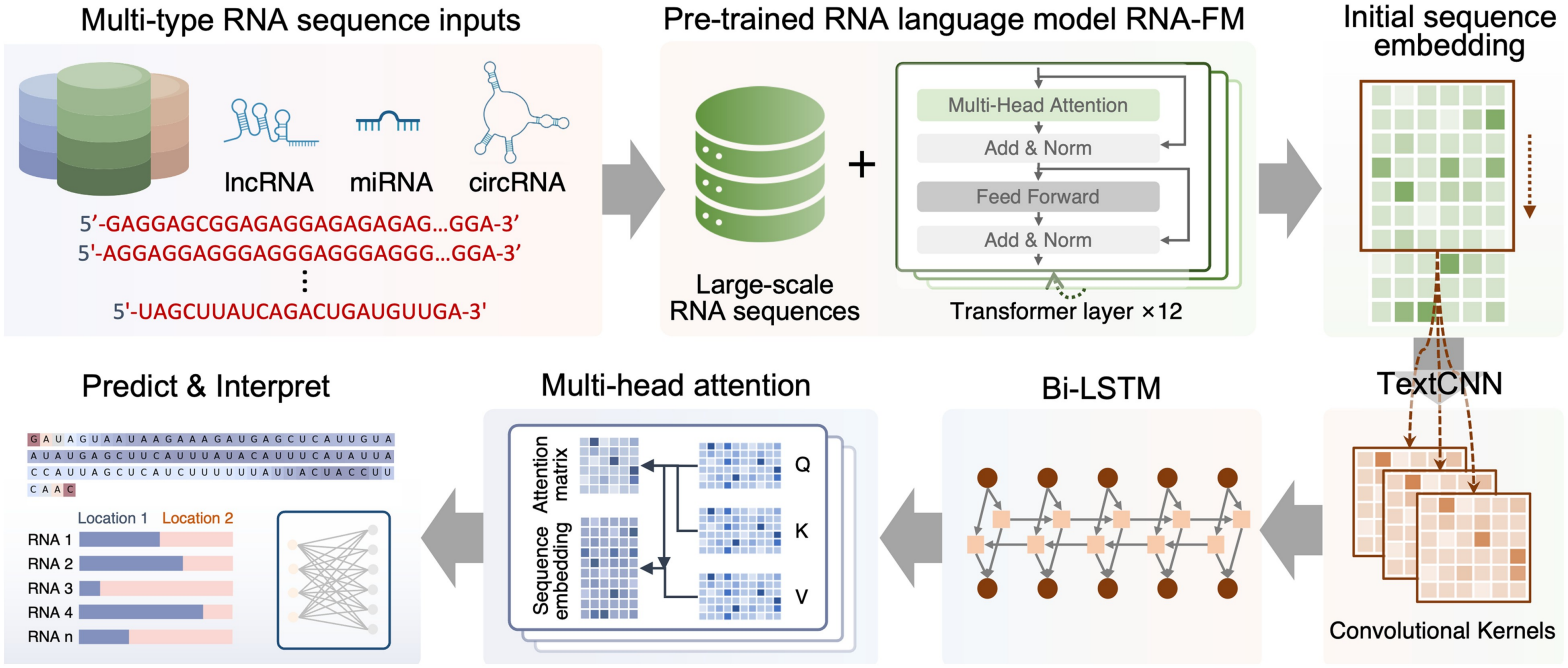
Architecture of RNALoc-LM. RNALoc-LM uses the pre-trained RNA language model RNA-FM as its embedding module. By inputting the RNA sequence into this embedding module, an embedding matrix is generated. The TextCNN network is then used to extract local patterns from the embeddings. Following this, a BiLSTM module captures long-range dependencies and contextual information. Additionally, RNALoc-LM incorporates a multi-head attention mechanism to focus on the important segments of the RNA sequence. Finally, a fully connected layer is used to perform the RNA subcellular localization prediction task.

As illustrated in Fig. 1, RNALoc-LM takes RNA sequences as input to predict their subcellular localization. The architecture of RNALoc-LM consists of two main modules: the embedding module and the downstream network architecture. In the embedding module, the RNA language model, RNA-FM, is used to encode the RNA sequences. The output from the embedding module is then fed into the downstream network architecture for RNA subcellular localization prediction. During the training process, we freeze the network parameters of the RNA language model and only train the downstream network architecture.

#### 2.2.1 Embedding module

The embedding module uses a pre-trained RNA language model, RNA-FM, which is an RNA foundation model. RNA-FM consists of 12 Transformer-based bidirectional encoder blocks, each comprising a 640D feedforward layer and a 20-head self-attention layer. RNA-FM is trained on 23 million sequences from the RNAcentral database using a self-supervised approach, allowing it to learn multi-dimension feature representations of RNA sequences.

We leverage RNA-FM to extract representations for RNA sequences. Specifically, RNA-FM generates RNA representations with dimensions of *L* × 640, where 640 represents the embedding dimension and L denotes the length of the RNA sequences.

By encoding RNA sequences using the RNA-FM model, we can utilize its ability to capture long-range dependencies and obtain rich representations that encompass the structural and functional properties of non-coding RNAs, as well as their evolutionary information. This facilitates more accurate predictions for downstream tasks.

#### 2.2.2 Downstream network architecture

The downstream network architecture primarily consists of a TextCNN module, a BiLSTM module, a multi-head attention mechanism, and fully connected layers.

First, RNALoc-LM uses the TextCNN network to capture local correlations within RNA sequences. For each input tensor of size *L* × 640, RNALoc-LM applies various convolution kernels with sizes of [*k*, *d*] (where *k* is 3, 4, 5, and *d* corresponds to the width of the embedding data, i.e. 640). Each convolution layer applies a kernel of size [*k*, *d*] to the input tensor, moving in the direction of the tensor height to capture *n*-gram features of different lengths. Subsequently, RNALoc-LM performs a feature squeezing operation in the channel direction and activates the feature map using the ReLU function. Since the feature maps generated by different convolution kernels may vary in length, RNALoc-LM unifies the feature length by taking the minimum length of all feature maps, thereby extracting important information from the RNA sequence.

In RNALoc-LM, a bidirectional LSTM (BiLSTM) network processes the sequence in both forward and backward directions to capture long-range dependencies and contextual information. The BiLSTM captures features both directions along the sequences, calculating hidden states and concatenating them to obtain a more comprehensive feature representation. The hidden state $h_{t}$ and cell state $c_{t}$ at each time step of the forward LSTM are computed as follows:

(1) $$i_{t}=\sigma (W_{ix}x_{t}+W_{ih}h_{t-1}+b_{i})$$

 

(2) $$f_{t}=\sigma (W_{fx}x_{t}+W_{fh}h_{t-1}+b_{f})$$

 

(3) $$o_{t}=\sigma (W_{ox}x_{t}+W_{oh}h_{t-1}+b_{o})$$

 

(4) $${\tilde{c}}_{t}=\sigma (W_{cx}x_{t}+W_{ch}h_{t-1}+b_{c})$$

 

(5) $$c_{t}=f_{t}⊙c_{t-1}+i_{t}⊙{\tilde{c}}_{t}$$

 

(6) $$h_{t}=o_{t}⊙{\tanh(c}_{t})$$

where $i_{t},\;f_{t}\;$and $o_{t}\;$are the states of the input gate, forget gate, and output gate, respectively. σ represents the sigmoid function, and ⊙ represents the element-wise product.

Similarly, the hidden state $h_{t}^{\prime }$ and cell state $c_{t}^{\prime }$ of each time step of the reverse LSTM can be calculated by similar formulas. The output of the BiLSTM is obtained by concatenating the hidden states from both LSTMs:

(7) $$y_{t}=[h_{t};h_{t}^{\prime }]$$

In recent years, attention mechanisms have been widely applied to focus on important features and enhance feature extraction. In RNALoc-LM, we use a multi-head attention mechanism to capture important parts of the sequence for subcellular localization prediction while also obtaining attention weights for RNA motif analysis, thereby improving the model’s interpretability.

Multi-head attention effectively merges multiple self-attention results, capturing relevant information from different subspaces. The calculation process of attention is as follows:

(8) $$\mathrm{Attention}\left(Q,\;K,\;V\right)=\mathrm{Softmax}(\frac{QK^{T}}{\sqrt{d^{\prime }}})V$$

where Q, K, V are the input tensor obtained through matrix transformations, and d' is the dimension of the input tensor.

Multi-head attention uses multiple queries, each focusing on different parts of the tensor, to calculate and select various pieces of information from the input tensor, which are then concatenated.

(9) $$\mathrm{Multi}-\mathrm{Head}\left(Q,\;K,\;V\right)=\mathrm{Concat}({\mathrm{head}}_{1},\ldots ,{\mathrm{head}}_{n})W^{O}$$

where ${\mathrm{head}}_{i}=\mathrm{Attention}(QW_{i}^{Q},KW_{i}^{K},VW_{i}^{V})$, and $W_{i}^{Q},W_{i}^{K},W_{i}^{V},W^{O}$ are learnable parameters. *n* is the number of heads.

Finally, we obtain a series of latent space matrix and attention weight matrix. The extracted RNA features are linearly transformed through a fully connected layer for subcellular localization prediction, while the attention weight matrix is utilized for RNA motif analysis.

### 2.3 Deep learning baseline models

In order to verify the effectiveness of RNALoc-LM, three deep-learning baseline models were designed for comparison.

1. RNA-FM + Transformer + MLP, this model uses RNA-FM to encode RNA sequences, and uses a Transformer architecture to capture complex dependencies and contextual information from the input. The output of the Transformer is then passed through a Multi-Layer Perceptron (MLP) for prediction.
2. one-hot + TextCNN + Bi-LSTM + MLP, this model uses one-hot encoding for RNA sequences, and applies TextCNN to capture different n-gram features. It uses Bi-LSTM to capture sequential information, and uses a MLP for prediction.
3. word2vec + TextCNN + Bi-LSTM + MLP, this model uses word2vec encoding for RNA sequences, and applies TextCNN to capture different n-gram features. It uses Bi-LSTM to capture sequential information, and uses a MLP for prediction.

### 2.4 Evaluation metrics

To evaluate the performance of RNALoc-LM with other computational methods, we used four evaluation metrics, including accuracy (ACC), macro precision, and macro recall, and macro F1.

(10) $${\mathrm{Precision}}_{i}=\frac{{TP}_{i}}{{TP}_{i}+{FP}_{i}}$$

 

(11) $$\mathrm{Macro}\;\mathrm{Precision}=\frac{1}{n}\sum_{i=1}^{n}{\mathrm{Precision}}_{i}$$

 

(12) $${\mathrm{Recall}}_{i}=\frac{{TP}_{i}}{{TP}_{i}+{FN}_{i}}$$

 

(13) $$\mathrm{Macro}\;\mathrm{Recall}=\frac{1}{n}\sum_{i=1}^{n}{\mathrm{Recall}}_{i}$$

 

(14) $$\mathrm{Macro}\;F1=\frac{1}{n}\sum_{i=1}^{n}\frac{2*{\mathrm{Precision}}_{i}*{\mathrm{Recall}}_{i}}{{\mathrm{Precision}}_{i}+{\mathrm{Recall}}_{i}}$$

where n is the number of subcellular localization categories, ${TP}_{i}$, ${FP}_{i}$ and ${FN}_{i}$ represent the true positive, false positive, and false negative of category i, respectively. ${\mathrm{Precision}}_{i}$ and ${\mathrm{Recall}}_{i}$ represent the precision and recall of category i.

### 2.5 Implementation details

RNALoc-LM is implemented using Pytorch. The sequence length is clipped to the maximum length of RNA-FM, which is 1024, before data embedding. The initial learning rate is set to 0.001, and the “ReduceLROnPlateau” scheduler is used to adjust the learning rate when the validation loss stops improving. The scheduler reduces the learning rate when a monitored metric (e.g. validation loss) ceases to show improvement, helping the model escape suboptimal performance and converge more efficiently. Specifically, the mode is set to “minimum,” the factor is set to 0.5 (reducing the learning rate by half), and the “patience” parameter is set to 20 epochs. The Adam optimizer is used with a learning rate of 0.005 and a weight decay of 1e-4 to prevent overfitting.

To measure the loss between the true label and the predicted probability, we used the Focal Loss function. The Focal Loss function was introduced by Lin (2017), and is particularly effective in scenarios with significant class imbalance, as it emphasizes hard-to-classify examples while down-weighting the contribution of easy-to-classify ones.

The standard cross-entropy loss is defined as:

(15) $$\mathrm{Cross}-\mathrm{Entropy}\;\mathrm{Loss}=-\sum_{0}^{c-1}y_{i}\;*\;\log\left(p_{i}\right)=-\log(p_{c})$$

where $y_{i}\;\in \{0,\;1\}$ is the true label and $p_{c}$ is the predicted probability for the true class. Focal Loss modifies this by adding a modulating factor ${\left(1-p_{c}\right)}^{\gamma }\;$and a weighting factor α:

(16) $$\mathrm{Focal}\;\mathrm{Loss}=-\alpha_{c}{\left(1-p_{c}\right)}^{\gamma }l\mathrm{og}(p_{c})$$

Here,$\;\alpha_{c}\;$is a balancing factor for class weights, which adjusts the importance of positive and negative examples. γ is the focusing parameter that reduces the loss contribution from easy examples and extends the range in which hard examples receive greater attention.

In this study, α is calculated as follows:

(17) $$w_{c}=\frac{N}{N_{c}}$$

 

(18) $$\alpha_{c}=\frac{w_{c}}{\sum_{i=0}^{c-1}w_{i}}$$

To stabilize the training process and prevent gradient-related challenges, gradient clipping is applied. This technique ensures that updates to the model parameters stay within a reasonable range. Specifically, if the gradient norm exceeds a threshold, the gradient is rescaled to a norm which is equal to the threshold. In our work, we set the gradient norm threshold at 1.0, above which the gradient is clipped.

Additionally, we provide details regarding the training time and computational resources required to train RNALoc-LM in Supplementary Table S1.

## 3 Results

### 3.1 Comparison with deep-learning baseline models

To evaluate the performance of RNALoc-LM with deep-learning baseline models, we used 5-fold cross-validation (CV). In this approach, the dataset was divided into five equal parts. For each fold, 80% of the data was used for training, while the remaining 20% was used for validation. This procedure was repeated five times, ensuring that each fold was used as the validation set once. Consequently, we obtained results of five validation sets. We calculated the average performance across these five folds to evaluate the model’s performance. As shown in Table 2, we can observe that RNALoc-LM consistently outperforms other deep-learning baseline models in terms of ACC and Macro F1 across different RNA types. The results clearly demonstrate the superiority of RNALoc-LM and highlight the advantages of its architectural design.

**Table 2.** Performance comparison of RNALoc-LM and deep-learning baseline models using 5-fold CV.^a^

RNA type	Deep-learning baseline model	ACC	Macro F1	Macro precision	Macro recall
lncRNA	RNA-FM + Transformer + MLP	0.649	0.491	**0.713**	0.551
	one-hot + TextCNN + Bi-LSTM + MLP	0.633	0.388	0.316	0.500
	word2vec + TextCNN + Bi-LSTM + MLP	0.633	0.388	0.316	0.500
	RNALoc-LM	**0.677**	**0.593**	0.596	**0.613**
miRNA	RNA-FM + Transformer + MLP	0.913	0.903	**0.901**	0.907
	one-hot + TextCNN + Bi-LSTM + MLP	0.878	0.860	0.869	0.854
	word2vec + TextCNN + Bi-LSTM + MLP	0.881	0.866	0.868	0.867
	RNALoc-LM	**0.913**	**0.903**	0.899	**0.909**
circRNA	RNA-FM + Transformer + MLP	0.795	0.787	0.792	0.785
	one-hot + TextCNN + Bi-LSTM + MLP	0.797	0.789	0.794	0.786
	word2vec + TextCNN + Bi-LSTM + MLP	0.802	0.795	0.799	0.793
	RNALoc-LM	**0.804**	**0.797**	**0.802**	**0.794**

a The best performance values are highlighted in bold.

### 3.2 Comparison with existing predictors

**Figure 2.**
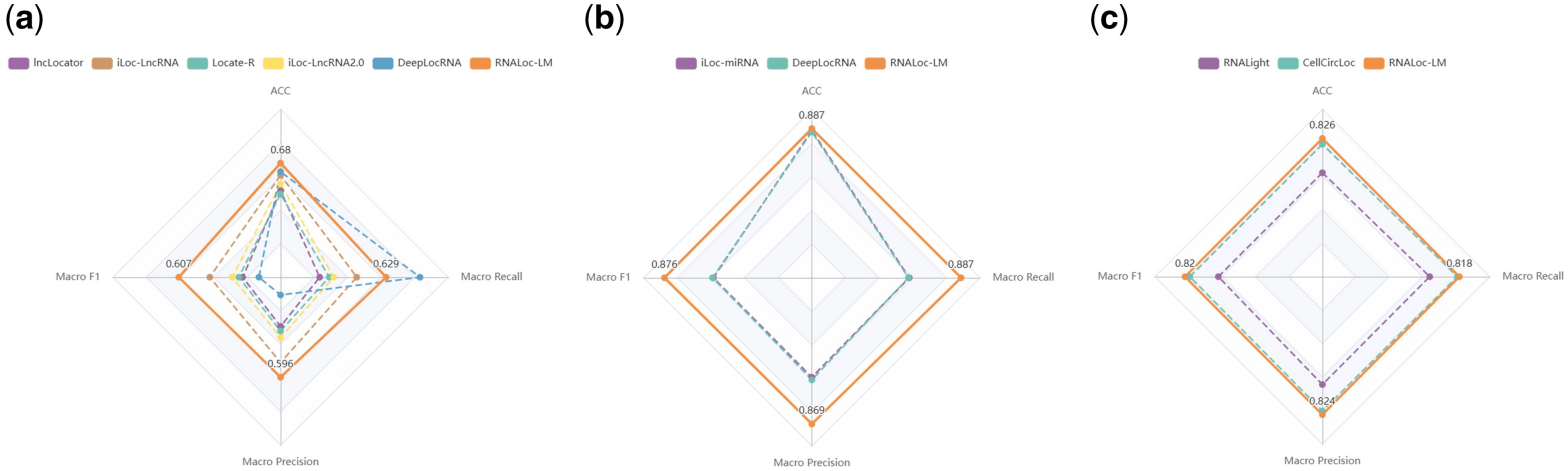
Performance comparison of RNALoc-LM with existing predictors on the independent test set. (a) Comparison of RNALoc-LM with five predictors for predicting lncRNA subcellular localization. (b) Comparison of RNALoc-LM with two predictors for predicting miRNA subcellular localization. (c) Comparison of RNALoc-LM with two predictors for predicting circRNA subcellular localization.

To further evaluate the performance of RNALoc-LM in predicting the subcellular localization of three types of RNAs, we conducted a comparison with several existing state-of-the-art predictors using independent test sets. The distribution of the independent test dataset can be seen in Supplementary Table S2. The criteria for selecting these predictors included: (i) availability of web servers or stand-alone versions; (ii) only requiring RNA sequences as input; (iii) output that includes predicted probabilities for subcellular localization. Specifically, for lncRNA, we compared RNALoc-LM with five predictors: lncLocator (http://www.csbio.sjtu.edu.cn/bioinf/lncLocator/), iLoc-lncRNA (http://lin-group.cn/server/iLoc-LncRNA/), Locate-R (http://locate-r.azurewebsites.net), iLoc-lncRNA(2.0) (http://lin-group.cn/server/iLoc-LncRNA(2.0)/), and DeepLocRNA (https://biolib.com/KU/DeepLocRNA/). For miRNA, we compared RNALoc-LM with two predictors: iLoc-miRNA (http://iloc-mirna.lin-group.cn/), and DeepLocRNA (https://biolib.com/KU/DeepLocRNA/). For circRNA, we compared RNALoc-LM with two predictors: RNALight (https://github.com/YangLab/RNAlight), and CellCircLoc (http://csuligroup.com:8000/cellcircloc). To ensure a fair comparison between RNALoc-LM (a single-label predictor) and DeepLocRNA (a multi-label predictor), we evaluated DeepLocRNA based on whether its set of predicted labels covered the true label. If the true label was included in the predicted set, the prediction was considered correct; otherwise, it was considered incorrect. We then computed evaluation metrics for both predictors under this framework. The performance of RNALoc-LM with these existing predictors for the three types of RNAs is illustrated in Fig. 2.

From Fig. 2a, it is evident that RNALoc-LM outperforms the other five predictors, achieving the highest ACC of 0.680 and a Macro F1 score of 0.607. From Fig. 2b, we can see that RNALoc-LM outperforms the other two predictors, with an ACC of 0.887 and a Macro F1 score of 0.876. From Fig. 2c, RNALoc-LM again shows superior performance, with an ACC of 0.826 and a Macro F1 score of 0.820 compared to the other two predictors. All results confirm the effectiveness of RNALoc-LM in predicting RNA subcellular localization. The detailed prediction results of RNALoc-LM and the other predictors on independent test sets for lncRNA, miRNA, and circRNA can be seen in Supplementary Tables S3–S5, respectively, while corresponding confusion matrices are shown in Supplementary Figs S1–S3.

### 3.3 Motif analysis

RNALoc-LM uses a multi-head attention mechanism to obtain attention weights at the nucleic acid level, enabling the identification of important motifs in RNA sequences. To demonstrate RNALoc-LM’s ability in capturing motifs, we conducted a series of motif analyses.

**Figure 3.**
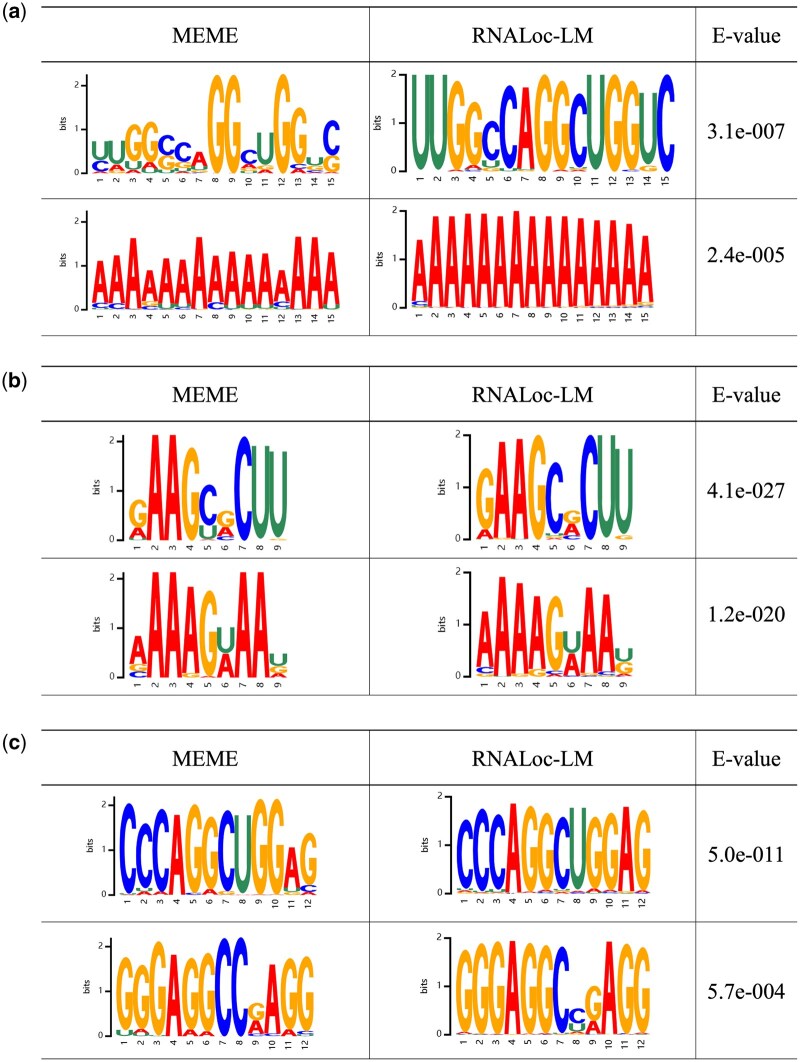
Motifs identified by the MEME suite (left) and by RNALoc-LM (middle). The right column displays the E-values for the motifs discovered by the MEME suite. (a) Identified lncRNA motifs. (b) Identified miRNA motifs. (c) Identified circRNA motifs.

First, we evaluated whether RNALoc-LM could identify the most frequently recurring motifs in the sequences. For this purpose, we used the MEME suite (Bailey *et al.* 2009) to discover motifs in our dataset, analyzing motifs with widths ranging from 9 to 15 and setting the E-value threshold at 0.05. In RNALoc-LM, we used a threshold for determining the importance of attention weights, defined as the median value of all attention weights in the sequence. A nucleotide is considered important if its attention weight exceeds the threshold. Figure 3 shows several representative motifs, the left column displays the motifs identified by MEME, the middle column shows the motifs discovered by RNALoc-LM, and the right column shows the E-values of the motifs discovered by MEME. From Fig. 3, it is evident that RNALoc-LM effectively captures motifs similar to those identified by the MEME suite across the three types of RNAs, demonstrating RNALoc-LM’s ability to identify the most frequently recurring motifs within RNA sequences.

**Figure 4.**
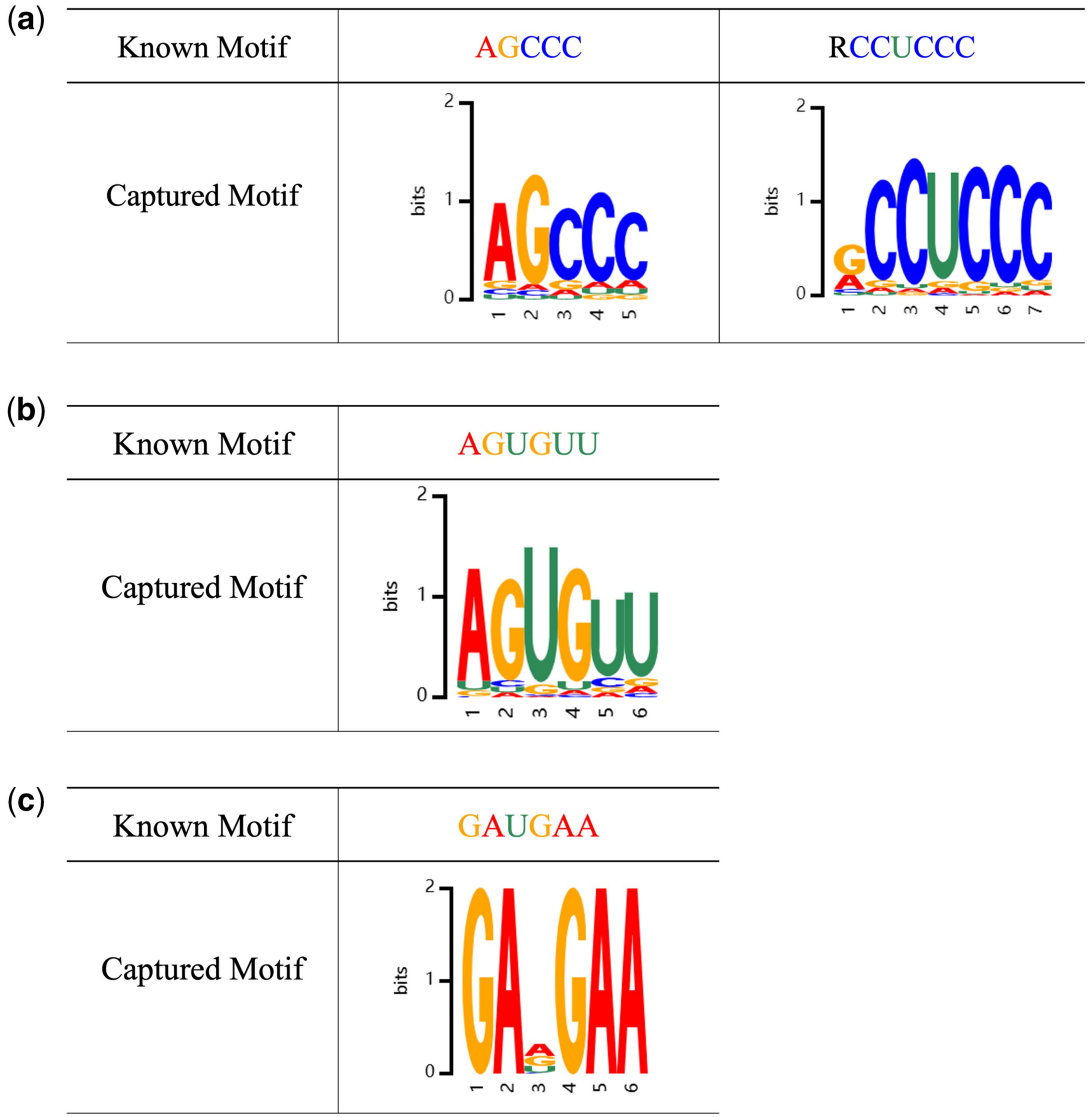
RNALoc-LM captures known motifs associated with subcellular localization in different RNA types. (a) RNALoc-LM captures the motifs “AGCCC” and “RCCUCCC,” both associated with nuclear localization in lncRNAs. (b) RNALoc-LM captures the “AGUGUU” motif in miRNAs, associated with nuclear localization. (c) RNALoc-LM captures the “GAUGAA” motif in circRNAs, which is associated with nucleus localization.

Second, we investigated whether RNALoc-LM could capture some experimentally validated motifs related to subcellular localization. Specifically, we searched recent literature for experimentally verified motifs associated with subcellular localization. For lncRNA, Lubelsky and Ulitsky (2018) found that the repetitive motif “RCCUCCC” (where R represents A/G) drives lncRNA localization to the nucleus. Additionally, Zhang *et al.* (2014) identified the motif “AGCCC” acts as a universal nuclear localization signal. We used motifs “RCCTCCC” and “AGCCC” as examples to demonstrate the performance of RNALoc-LM in capturing known lncRNA subcellular localization related motifs, as shown in Fig. 4a. For miRNA, Hwang *et al.* (2007) found that the motif “AGUGUU” is associated with nuclear localization, including its variants “UGUGUU,” “AGAGUU,” “ACUCUU,” “AGUGAU,” “AGUGUA,” and “AGNGUN.” We used motif “AGUGUU” as an example to illustrate the performance of RNALoc-LM in capturing known miRNA subcellular localization related motifs, as shown in Fig. 4b. For circRNA, Barbagallo *et al.* (2021) identified the motif “GAUGAA” is an experimentally validated SRSF1 binding motif, noting that circRNA containing SRSF1 binding motifs were enriched in the nuclear fraction. We used motif “GAUGAA” as an example to demonstrate the performance of RNALoc-LM in capturing known circRNA subcellular localization related motifs, as illustrated in Fig. 4c. From Fig. 4, it is clear that RNALoc-LM can effectively capture motifs that closely resemble those previously validated experimentally.

### 3.4 Case study

**Figure 5.**
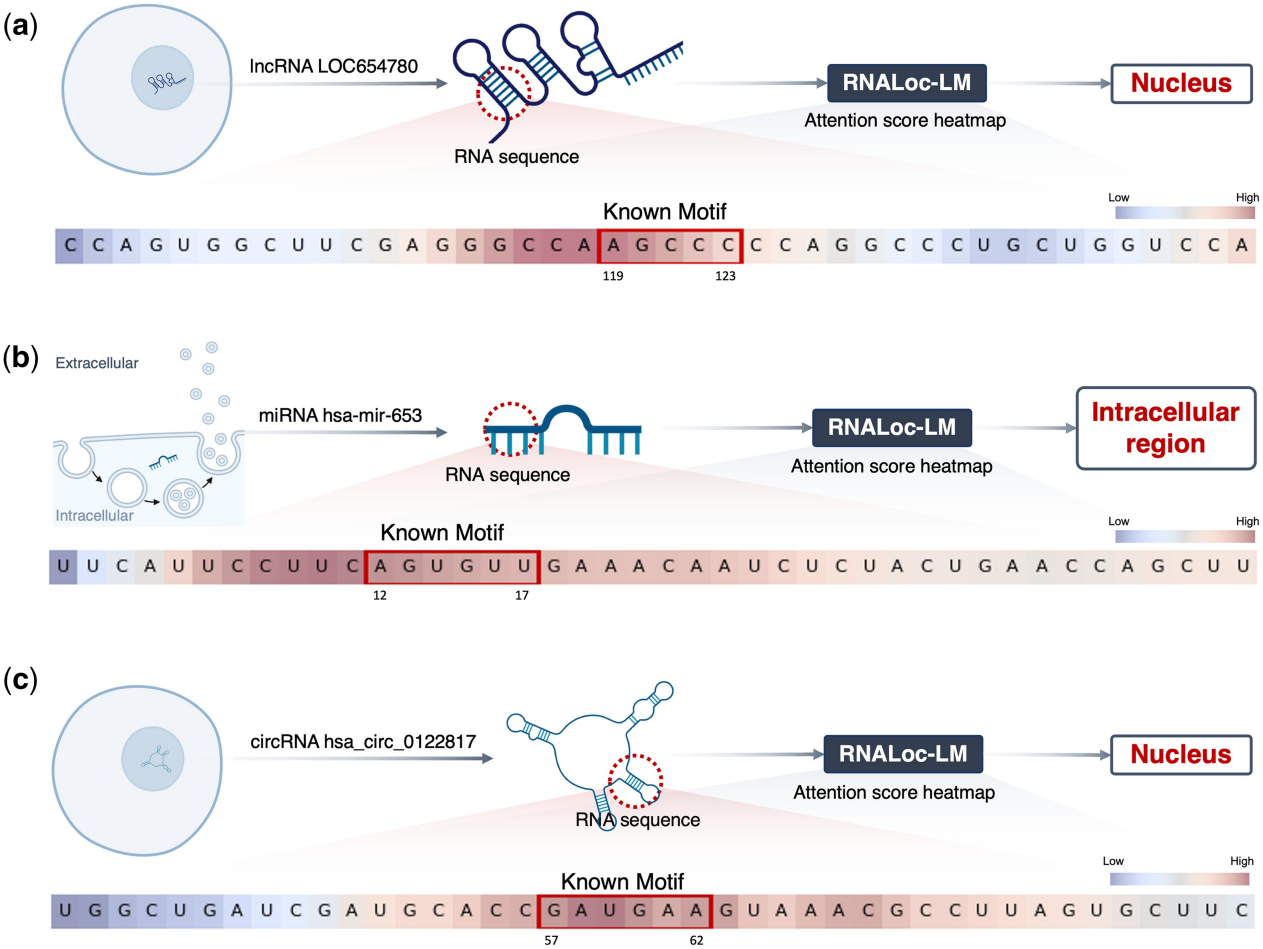
Prediction results of three representative RNA samples from using RNALoc-LM. (a) Prediction results for lncRNA LOC654780. (b) Prediction results for miRNA hsa-mir-653. (c) Prediction results for circRNA hsa_circ_0122817.

To better understand the mechanism of RNALoc-LM, we selected a representative sample from each type of RNA as a case study to demonstrate the ability of RNALoc-LM. For lncRNA, we took lncRNA LOC654780 (NCBI ID: 654780) as an example. Its true label is the nucleus, and RNALoc-LM predict its subcellular localization as the nucleus. Additionally, RNALoc-LM highlights the motif “AGCCC” in its sequence, as shown in Fig. 5a. For miRNA, we took miRNA hsa-mir-653 (NCBI ID: 724023) as an example. Its true label is also the nucleus, and RNALoc-LM predict its subcellular localization as the intracellular region. In addition, RNALoc-LM highlights the motif “AGUGUU” in its sequence, as illustrated in Fig. 5b. For circRNA, we took circRNA hsa_circ_0122817 (circBase ID: hsa_circ_0122817) as an example. Its true label for the circRNA is the nucleus, and RNALoc-LM predict its subcellular localization as the nucleus as well. RNALoc-LM highlights the motif “GAUGAA” in its sequence, as shown in Fig. 5c. These case studies highlight RNALoc-LM’s effectiveness in accurately predicting RNA subcellular localization and its ability to capture important motifs within RNA sequences.

### 3.5 Ablation study

To evaluate the contribution of each component of RNALoc-LM, we conducted an ablation study. We designed five variant models: one without the RNA language model (replaced with one-hot coding), one without the RNA language model (replaced with word2vec coding), one without the CNN module, one without the Bi-LSTM module, and one without the attention mechanism. The performance of RNALoc-LM and its variant models was evaluated on independent test sets for three types of RNAs.

As shown in Table 3, we observed that (i) all modules positively contribute to RNA subcellular localization prediction tasks. Taking lncRNA as an example, using one-hot coding replace the RNA language model, ACC decreased from 0.680 to 0.641, and macro F1 decreased from 0.607 to 0.418. Using word2vec coding replace the RNA language model, ACC decreased from 0.680 to 0.579, and macro F1 decreased from 0.607 to 0.418. Without the CNN module, ACC decreased to 0.660, and macro F1 decreased to 0.579. Without the Bi-LSTM module, ACC decreased to 0.674, and macro F1 decreased to 0.571. Without the attention mechanism, ACC decreased to 0.641, and macro F1 decreased to 0.506. (ii) The removal of the RNA language model had the most significant impact on performance. For lncRNA, using one-hot coding replace the RNA language model, ACC decreased from 0.680 to 0.641, and macro F1 decreased from 0.607 to 0.418. Using word2vec coding replace the RNA language model, ACC decreased from 0.680 to 0.579, and macro F1 decreased from 0.607 to 0.418. For miRNA, using one-hot coding replace the RNA language model, ACC decreased from 0.887 to 0.839, and macro F1 decreased from 0.876 to 0.776. Using word2vec coding replace the RNA language model, ACC decreased from 0.887 to 0.867, macro F1 decreased from 0.876 to 0.846. For circRNA, using one-hot coding replace the RNA language model, ACC decreased from 0.826 to 0.797, and macro F1 decreased from 0.820 to 0.788. Using word2vec coding replace the RNA language model, ACC decreased from 0.826 to 0.640, macro F1 decreased from 0.820 to 0.557. The results across all RNA types confirm the essential role of the RNA language model in enhancing the model’s predictive capabilities.

**Table 3.** Performance of RNALoc-LM and its variant models on independent test sets for three types of RNAs.^a^

RNA type	Variant model	ACC	Macro F1	Macro precision	Macro recall
lncRNA	Without RNA-FM (one-hot)	0.641	0.418	0.376	0.510
	Without RNA-FM (word2vec)	0.579	0.418	0.385	0.494
	Without CNN	0.660	0.579	0.576	0.608
	Without Bi-LSTM	0.674	0.571	0.585	0.612
	Without attention	0.641	0.506	0.489	0.554
	RNALoc-LM	**0.680**	**0.607**	**0.596**	**0.629**
miRNA	Without RNA-FM (one-hot)	0.839	0.776	0.760	0.801
	Without RNA-FM (word2vec)	0.867	0.846	0.858	0.852
	Without CNN	0.869	0.855	0.851	0.862
	Without Bi-LSTM	0.885	0.874	0.867	0.884
	Without attention	0.880	0.868	0.862	0.877
	RNALoc-LM	**0.887**	**0.876**	**0.869**	**0.887**
circRNA	Without RNA-FM (one-hot)	0.797	0.788	0.797	0.784
	Without RNA-FM (word2vec)	0.640	0.557	0.635	0.617
	Without CNN	0.820	0.815	0.816	0.814
	Without Bi-LSTM	0.812	0.805	0.809	0.803
	Without attention	0.800	0.794	0.796	0.793
	RNALoc-LM	**0.826**	**0.820**	**0.824**	**0.818**

a The best performance values are highlighted in bold.

### 3.6 Web server

To facilitate the use of RNALoc-LM, we developed a user-friendly web server at http://csuligroup.com:8000/RNALoc-LM. The RNALoc-LM web server allows users to input up to ten sequences of lncRNA, miRNA, and circRNA in FASTA format and obtain predicted subcellular localization results by clicking the “submit” button. This user-friendly web server provides researchers and users with a convenient way to obtain the subcellular localization prediction results for their RNAs of interest.

## 4 Conclusion

RNA subcellular localization provides valuable insights into RNA functions, making the prediction of RNA subcellular localization critical. In recent years, RNA language models have demonstrated promising potential in various RNA bioinformatics tasks. Inspired by these advancements, we propose a novel deep-learning model, RNALoc-LM, which leverages a pre-trained RNA language model to predict the subcellular localizations of multiple types of RNAs. RNALoc-LM uses the RNA-FM model to encode RNA sequences, utilizing TextCNN and BiLSTM modules to extract local features and capture long-range dependencies. Furthermore, a multi-head attention mechanism is incorporated to focus on important segments of the RNA sequence. The results demonstrate that RNALoc-LM achieves superior performance in predicting the subcellular localization of three types of RNAs: lncRNA, circRNA, and miRNA, consistently outperforming existing state-of-the-art predictors.

Despite these promising results of RNALoc-LM, certain limitations warrant further improvement. First, the performance of RNALoc-LM is constrained by the number of available samples. In the study, we collected 1003 lncRNAs, 1124 miRNAs, 27 682 circRNAs. While the number of circRNA is relatively large, the numbers of lncRNA and miRNA are limited. As RNA subcellular localization continues to gain importance as a research topic, acquiring more reliable data for training and testing will be essential. Second, RNALoc-LM currently classifies subcellular localizations into only two categories (nucleus and cytoplasm for lncRNAs and circRNAs, extracellular region and intracellular region for miRNAs). Although these categories are of primary interest to biologists, there is potential to classify a broader range of subcellular localizations. Third, RNALoc-LM focuses only on RNAs associated with a single subcellular localization. However, many RNAs can localize to multiple subcellular localizations in reality. Future work will aim to address this limitation by using a multi-label perspective to better predict RNAs with multiple subcellular localizations.

## Author contributions

Min Zeng (Conceptualization, Investigation, Formal analysis, Methodology, Project administration, Funding acquisition, Supervision, Writing—original draft, Writing—review & editing), Xinyu Zhang (Data curation, Formal analysis, Methodology, Validation, Software, Writing—original draft, Writing—review & editing), Yiming Li (Formal analysis, Visualization, Software, Writing—original draft, Writing—review & editing), Chengqian Lu (Conceptualization, Writing—review & editing), Rui Yin (Conceptualization, Writing—review & editing), Fei Guo (Conceptualization, Writing—review & editing), Min Li (Conceptualization, Supervision, Project administration, Writing—review & editing)

## Supplementary Material

btaf127_Supplementary_Data

## Data Availability

All data used in this study are available at https://github.com/CSUBioGroup/RNALoc-LM.
