# Supplementary material for: RNALoc-LM: RNA subcellular localization prediction using pre-trained RNA language model
Source: Bioinformatics. 2025 Mar 22;41(4):btaf127. doi: 10.1093/bioinformatics/btaf127 (PMC11978386; doi:10.1093/bioinformatics/btaf127)
Supplement: btaf127_Supplementary_Data [file btaf127_supplementary_data.zip › Supplementary materials20250228.pdf]

Supplementary materials:

RNALoc-LM: RNA subcellular localization prediction using pre-trained RNA language model

Min Zeng[1], Xinyu Zhang[1], Yiming Li[1], Chengqian Lu[2], Rui Yin[3], Fei Guo[1], Min Li[1, *]

[1]School of Computer Science and Engineering, Central South University, Changsha, 410083, China

[2]School of Computer Science, Key Laboratory of Intelligent Computing and Information Processing, Xiangtan University, Xiangtan, Hunan, 411105, China

[3]Department of Health Outcomes and Biomedical Informatics, University of Florida, Gainesville, Florida, 32603, USA

* Correspondence to: Min Li, E-mail: limin@mail.csu.edu.cn

This supplementary file includes:

1. Supplementary Figures

Supplementary Figure S1. Confusion matrices of RNALoc-LM with other existing predictors on the lncRNA test set. (a) lncLocator, (b) iLoc-lncRNA, (c) Locate-R, (d) iLoc-lncRNA 2.0, (e) DeepLocRNA, (f) RNALoc-LM.
Supplementary Figure S2. Confusion matrices of RNALoc-LM with other existing predictors on the miRNA test set. (a) iLoc-miRNA, (b) DeepLocRNA, (c) RNALoc-LM.
Supplementary Figure S3. Confusion matrices of RNALoc-LM with other existing predictors on the circRNA test set. (a) RNALight, (b) CellCircLoc, (c) RNALoc-LM.

2. Supplementary Tables

Supplementary Table S1. Computational resources, training time, batch size, and epochs used by different types of RNA training processes.
Supplementary Table S2. Distribution of the independent test dataset.
Supplementary Table S3. The detailed prediction results of lncLocator, iLoc-LncRNA, Locate-R, iLoc-LncRNA 2.0, DeepLocRNA, and RNALoc-LM on the lncRNA independent test set.
Supplementary Table S4. The detailed prediction results of RNALight, CellCircLoc, and RNALoc-LM on the miRNA independent test set.
Supplementary Table S5. The detailed prediction results of iLoc-miRNA, DeepLocRNA, and RNALoc-LM on the circRNA independent test set.

1. Supplementary Figures

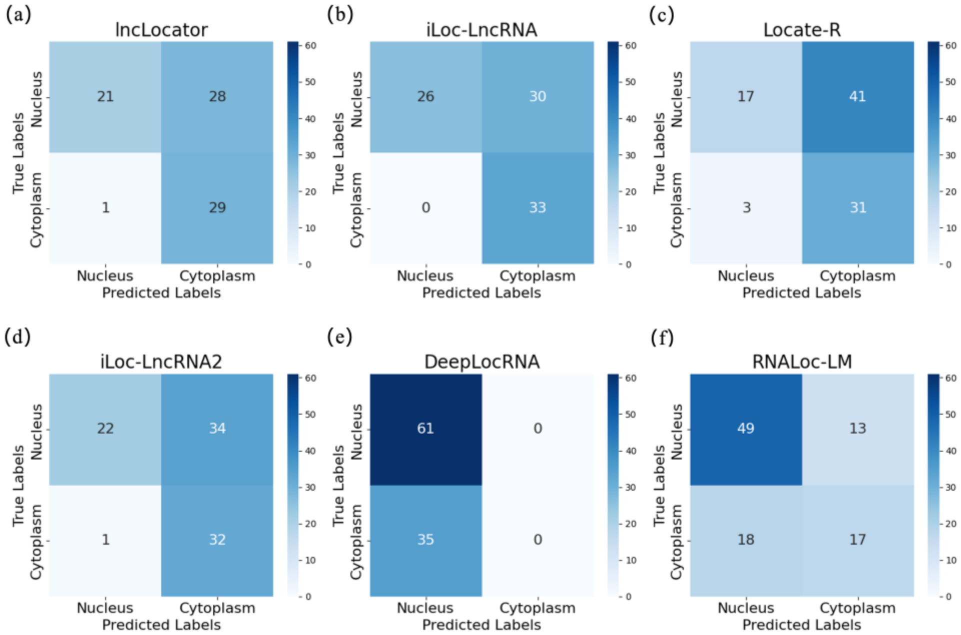

Supplementary Figure S1. Confusion matrices of RNALoc-LM with other existing predictors on the lncRNA independent test set. (a) lncLocator, (b) iLoc-lncRNA, (c) Locate-R, (d) iLoc-lncRNA 2.0, (e) DeepLocRNA, (f) RNALoc-LM.

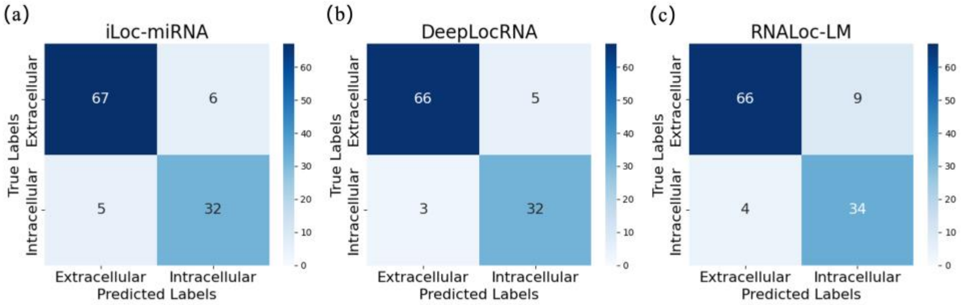

Supplementary Figure S2. Confusion matrices of RNALoc-LM with other existing predictors on the miRNA test set. (a) iLoc-miRNA, (b) DeepLocRNA, (c) RNALoc-LM.

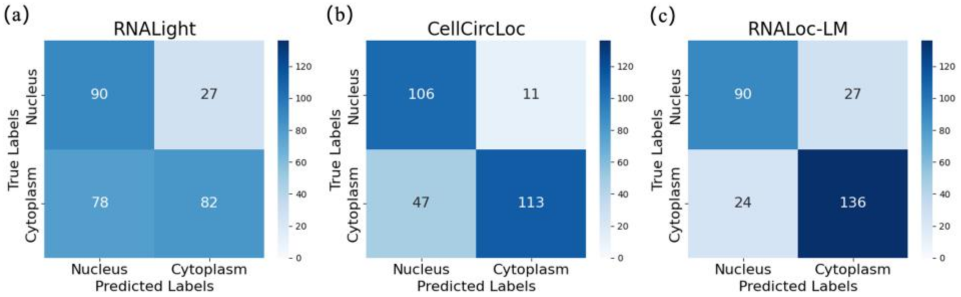

Supplementary Figure S3. Confusion matrices of RNALoc-LM with other existing predictors on the circRNA test set. (a) RNALight, (b) CellCircLoc, (c) RNALoc-LM.

2. Supplementary Tables

Supplementary Table S1. Computational resources, training time, batch size, and epochs used by different types of RNA training processes.

RNA Type	GPU	Time	Batch Size	Epochs
lncRNA	1 NVIDIA RTX A6000	29min	256	80
miRNA	2 NVIDIA GeForce RTX 2080 Ti	17min	64	40
circRNA	2 NVIDIA GeForce RTX 2080 Ti	10h43min	32	60

Supplementary Table S2. Distribution of the independent test dataset.

RNA Type	Subcellular localization	# of samples
lncRNA	Cytoplasm	35
	Nucleus	62
miRNA	Extracellular region	38
	Intracellular region	75
circRNA	Cytoplasm	160
	Nucleus	117

Supplementary Table S3. The detailed prediction results of lncLocator, iLoc-LncRNA, Locate-R, iLoc-LncRNA 2.0, DeepLocRNA, and RNALoc-LM on the lncRNA independent test set.

No.	lncRNA	lncLocator	iLoc-LncRNA	Locate-R	iLoc-LncRNA 2.0	DeepLocRNA	RNALoc-LM	True Labels
1	>ID|6763|gene_id|213409|transcript_id|NM_001033250 Lemd1 Mus musculus lncRNA	Cytoplasm	Cytoplasm, Cytosol	Cytoplasm	Cytoplasm, Cytosol	Nucleus	Nucleus	Cytoplasm
2	>ID|29917|gene_id|AK139316|transcript_id|AK139316 AK139316 Mus musculus lncRNA	Nucleus	Nucleolus, Nucleus, Nucleoplasm	Nucleus	Nucleolus, Nucleus, Nucleoplasm	Nucleus/Exosome	Cytoplasm	Nucleus
3	>ID|29899|gene_id|AK133692|transcript_id|AK133692 AK133692 Mus musculus lncRNA	Cytoplasm	Cytoplasm, Cytosol	Cytoplasm	Cytoplasm, Cytosol	Nucleus	Cytoplasm	Cytoplasm
4	>ID|56437|gene_id|ENSG00000254694|transcript_id|ENST00000532866 RP11-50B3.4 Homo sapiens lncRNA	Nucleus	Nucleolus, Nucleus, Nucleoplasm	Cytoplasm	Nucleolus, Nucleus, Nucleoplasm	None	Nucleus	Nucleus
5	>NCBI:284739|LINC00176|C20orf204|Homo sapiens lncRNA	Cytosol	Cytoplasm, Cytosol	Cytoplasm	Cytoplasm, Cytosol	Nucleus/Exosome	Nucleus	Nucleus
6	>ID|56209|gene_id|ENSG00000253476|transcript_id|ENST00000523840 RP11-395I14.2 Homo sapiens lncRNA	Cytoplasm	Cytoplasm, Cytosol	Cytoplasm	Cytoplasm, Cytosol	Nucleus	Nucleus	Nucleus
7	>NCBI:654780|lincSFPQ| LOC654780| Homo sapiens lncRNA	Cytosol	Ribosome	Cytoplasm	Nucleolus, Nucleus, Nucleoplasm	Nucleus/Exosome	Nucleus	Nucleus
8	>ID|56118|gene_id|ENSG00000225889|transcript_id|ENST00000438115 AC074289.1 Homo sapiens lncRNA	Cytoplasm	Cytoplasm, Cytosol	Nucleus	Cytoplasm, Cytosol	Nucleus/Exosome	Nucleus	Nucleus
9	>ID|56375|gene_id|ENSG00000251259|transcript_id|ENST00000504082 AC004069.2 Homo sapiens lncRNA	Cytoplasm	Nucleolus, Nucleus, Nucleoplasm	Cytoplasm	Nucleolus, Nucleus, Nucleoplasm	Nucleus	Cytoplasm	Nucleus
10	>ID|56665|gene_id|ENSG00000255318|transcript_id|ENST00000533311 RP11-655M14.13 Homo sapiens lncRNA	Cytoplasm	Cytoplasm, Cytosol	Exosome	Cytoplasm, Cytosol	Nucleus/Exosome/ Membrane	Nucleus	Nucleus
11	>ID|18245|gene_id|545486|transcript_id|NM_001080971 Tubb1 Mus musculus lncRNA	Cytoplasm	Cytoplasm, Cytosol	Cytoplasm	Cytoplasm, Cytosol	Nucleus/Exosome	Nucleus	Cytoplasm
12	>NCBI:100507463|PSMB8-AS1|PSMB8-AS1|Homo sapiens lncRNA	Cytosol	Cytoplasm, Cytosol	Cytoplasm	Cytoplasm, Cytosol	Nucleus	Nucleus	Cytoplasm
13	>NCBI:101978719|LINC00970|LINC00970|Homo sapiens lncRNA	Cytoplasm	Cytoplasm, Cytosol	Cytoplasm	Cytoplasm, Cytosol	Nucleus/Cytosol	Nucleus	Nucleus
14	>ID|56119|gene_id|ENSG00000234913|transcript_id|ENST00000441167 XXbac-B476C20.13 Homo sapiens lncRNA	Ribosome	Ribosome	Ribosome	Ribosome	Nucleus/Cytosol	Nucleus	Cytoplasm
15	>ID|22712|gene_id|67501|transcript_id|NM_001025615 Ccdc50 Mus musculus lncRNA	Nucleus	Nucleolus, Nucleus, Nucleoplasm	Cytoplasm	Cytoplasm, Cytosol	Nucleus/Exosome/ Cytosol/Membrane	Nucleus	Nucleus
16	>ID|825|gene_id|102631772|transcript_id|XR_381746 Gm30025 Mus musculus lncRNA	Cytoplasm	Ribosome	Cytoplasm	Ribosome	Nucleus/Exosome	Nucleus	Cytoplasm
17	>NCBI:78998|RHPN1-AS1|RHPN1-AS1| Homo sapiens lncRNA	Cytosol	Cytoplasm, Cytosol	Cytoplasm	Cytoplasm, Cytosol	Nucleus/Exosome/ Cytosol/Membrane	Nucleus	Cytoplasm
18	>NCBI:100506965|PWAR6|PWAR6|Homo sapiens lncRNA	Cytoplasm	Nucleolus, Nucleus, Nucleoplasm	Cytoplasm	Cytoplasm, Cytosol	Nucleus/Exosome/ Membrane	Cytoplasm	Nucleus
19	>NCBI:100874028|SGO1-AS1|SGO1-AS1|Homo sapiens lncRNA	Cytoplasm	Cytoplasm, Cytosol	Nucleus	Cytoplasm, Cytosol	Nucleus	Nucleus	Nucleus
20	>ID|29947|gene_id|AK156672|transcript_id|AK156672 AK156672 Mus musculus lncRNA	Cytoplasm	Cytoplasm, Cytosol	Cytoplasm	Cytoplasm, Cytosol	Nucleus	Nucleus	Cytoplasm
21	>ID|56037|gene_id|ENSG00000226310|transcript_id|ENST00000439622 RP3-323P24.3 Homo sapiens lncRNA	Cytoplasm	Cytoplasm, Cytosol	Cytoplasm	Cytoplasm, Cytosol	Nucleus/Exosome/ Membrane	Nucleus	Nucleus
22	>NCBI:221178|SPATA13|SPATA13|Homo sapiens lncRNA	Cytosol	Cytoplasm, Cytosol	Nucleus	Nucleolus, Nucleus, Nucleoplasm	Nucleus/Exosome/ Cytosol/Membrane	Nucleus	Nucleus
23	>NCBI:101927380|C9orf41-AS1|C9orf41-AS1|Homo sapiens lncRNA	Cytoplasm	Cytoplasm, Cytosol	Cytoplasm	Cytoplasm, Cytosol	Nucleus/Exosome/ Membrane	Nucleus	Nucleus
24	>ID|29937|gene_id|AK144201|transcript_id|AK144201 AK144201 Mus musculus lncRNA	Cytoplasm	Cytoplasm, Cytosol	Cytoplasm	Cytoplasm, Cytosol	Nucleus/Exosome	Nucleus	Cytoplasm
25	>ID|29886|gene_id|AK089620|transcript_id|AK089620 AK089620 Mus musculus lncRNA	Cytoplasm	Cytoplasm, Cytosol	Cytoplasm	Cytoplasm, Cytosol	Nucleus	Nucleus	Cytoplasm
26	>ID|6909|gene_id|216459|transcript_id|NM_172259 Myl6b Mus musculus lncRNA	Nucleus	Cytoplasm, Cytosol	Cytoplasm	Exosome	Nucleus/Exosome	Nucleus	Nucleus
27	>ID|30057|gene_id|BC075654|transcript_id|BC075654 BC075654 Mus musculus lncRNA	Cytoplasm	Cytoplasm, Cytosol	Cytoplasm	Cytoplasm, Cytosol	Nucleus	Cytoplasm	Cytoplasm
28	>ID|56378|gene_id|ENSG00000253865|transcript_id|ENST00000521756 RP11-394O4.3 Homo sapiens lncRNA	Cytosol	Cytoplasm, Cytosol	Cytoplasm	Cytoplasm, Cytosol	Nucleus/Cytosol	Cytoplasm	Nucleus
29	>ID|56480|gene_id|ENSG00000255893|transcript_id|ENST00000541092 RP11-685N10.1 Homo sapiens lncRNA	Cytoplasm	Cytoplasm, Cytosol	Cytoplasm	Cytoplasm, Cytosol	Nucleus	Cytoplasm	Nucleus
30	>NCBI:100499177|THAP9-AS1|THAP9-AS1| Homo sapiens lncRNA	Nucleus	Nucleolus, Nucleus, Nucleoplasm	Cytoplasm	Nucleolus, Nucleus, Nucleoplasm	Nucleus/Exosome/ Membrane	Nucleus	Nucleus
31	>ID|56306|gene_id|ENSG00000230551|transcript_id|ENST00000499521 CTB-89H12.4 Homo sapiens lncRNA	Cytoplasm	Cytoplasm, Cytosol	Cytoplasm	Cytoplasm, Cytosol	Nucleus/Exosome/ Membrane	Cytoplasm	Cytoplasm
32	>ID|29945|gene_id|AK156229|transcript_id|AK156229 AK156229 Mus musculus lncRNA	Cytoplasm	Cytoplasm, Cytosol	Cytoplasm	Cytoplasm, Cytosol	Nucleus	Cytoplasm	Cytoplasm
33	>ID|56313|gene_id|ENSG00000250081|transcript_id|ENST00000510261 CTD-2116N20.1 Homo sapiens lncRNA	Cytoplasm	Cytoplasm, Cytosol	Cytoplasm	Cytoplasm, Cytosol	Nucleus	Nucleus	Nucleus
34	>ID|56300|gene_id|ENSG00000250453|transcript_id|ENST00000504398 CTD-2134P3.1 Homo sapiens lncRNA	Cytoplasm	Cytoplasm, Cytosol	Cytoplasm	Cytoplasm, Cytosol	Nucleus/Exosome/ Membrane	Nucleus	Nucleus

35	>ID|56166|gene_id|ENSG00000248774|transcript_id|ENST00000507803 RP11-798M19.3 Homo sapiens lncRNA	Cytoplasm	Nucleolus, Nucleus, Nucleoplasm	Cytoplasm	Cytoplasm, Cytosol	Nucleus/Exosome/ Cytosol/Membrane	Cytoplasm	Nucleus
36	>ID|55947|gene_id|ENSG00000238221|transcript_id|ENST00000379928 RP11-69L16.4 Homo sapiens lncRNA	Cytosol	Cytoplasm, Cytosol	Cytoplasm	Cytoplasm, Cytosol	Nucleus/Exosome/ Cytosol/Membrane	Nucleus	Nucleus
37	>ID|315|gene_id|100503470|transcript_id|XR_105783 Gm16619 Mus musculus lncRNA	Cytoplasm	Cytoplasm, Cytosol	Cytoplasm	Cytoplasm, Cytosol	Nucleus	Cytoplasm	Cytoplasm
38	>NCBI:101928969|RHOXF1-AS1|RHOXF1-AS1|Homo sapiens lncRNA	Cytoplasm	Cytoplasm, Cytosol	Cytoplasm	Cytoplasm, Cytosol	Nucleus	Nucleus	Nucleus
39	>ID|56325|gene_id|ENSG00000231856|transcript_id|ENST00000456688 RP11-327P2.5 Homo sapiens lncRNA	Cytoplasm	Cytoplasm, Cytosol	Nucleus	Cytoplasm, Cytosol	Nucleus/Exosome/ Cytosol/Membrane	Nucleus	Nucleus
40	>NCBI:348021|LINC00442 |LINC00442|Homo sapiens lncRNA	Cytoplasm	Nucleolus, Nucleus, Nucleoplasm	Cytoplasm	Cytoplasm, Cytosol	Nucleus	Cytoplasm	Nucleus
41	>ID|30603|gene_id|X53630|transcript_id|X53630 X53630 Mus musculus lncRNA	Cytoplasm	Cytoplasm, Cytosol	Cytoplasm	Cytoplasm, Cytosol	Nucleus	Nucleus	Cytoplasm
42	>ID|29790|gene_id|AK032710|transcript_id|AK032710 AK032710 Mus musculus lncRNA	Nucleus	Nucleolus, Nucleus, Nucleoplasm	Nucleus	Nucleolus, Nucleus, Nucleoplasm	Nucleus/Exosome	Cytoplasm	Nucleus
43	>NCBI:401164|LINC01060 |LINC01060|Homo sapiens lncRNA	Cytoplasm	Cytoplasm, Cytosol	Cytoplasm	Cytoplasm, Cytosol	Nucleus	Nucleus	Nucleus
44	>ID|1796|gene_id|107767|transcript_id|NM_029153 Scamp1 Mus musculus lncRNA	Cytoplasm	Cytoplasm, Cytosol	Nucleus	Cytoplasm, Cytosol	Nucleus/Exosome/ Membrane	Nucleus	Cytoplasm
45	>ID|11692|gene_id|319626|transcript_id|NR_015610 9530059O14Rik Mus musculus lncRNA	Nucleus	Nucleolus, Nucleus, Nucleoplasm	Nucleus	Nucleolus, Nucleus, Nucleoplasm	Nucleus/Exosome/ Membrane	Nucleus	Nucleus
46	>ID|29820|gene_id|AK044373|transcript_id|AK044373 AK044373 Mus musculus lncRNA	Cytoplasm	Cytoplasm, Cytosol	Cytoplasm	Cytoplasm, Cytosol	Nucleus	Cytoplasm	Cytoplasm
47	>NCBI:100874211|MID1IP1-AS1|MID1IP1-AS1|Homo sapiens lncRNA	Cytoplasm	Cytoplasm, Cytosol	Cytoplasm	Exosome	Nucleus	Nucleus	Nucleus
48	>ID|12096|gene_id|329271|transcript_id|NR_046171 C230024C17Rik Mus musculus lncRNA	Cytoplasm	Cytoplasm, Cytosol	Cytoplasm	Cytoplasm, Cytosol	Nucleus	Cytoplasm	Cytoplasm
49	>ID|1435|gene_id|105243929|transcript_id|XR_865436 Gm39644 Mus musculus lncRNA	Cytoplasm	Cytoplasm, Cytosol	Nucleus	Cytoplasm, Cytosol	Nucleus	Nucleus	Cytoplasm
50	>NCBI:107986136|IL20RB-AS1|IL20RB-AS1|Homo sapiens lncRNA	Cytoplasm	Nucleolus, Nucleus, Nucleoplasm	Cytoplasm	Cytoplasm, Cytosol	Nucleus	Nucleus	Nucleus
51	>ID|56650|gene_id|ENSG00000255176|transcript_id|ENST00000530198 AP002954.3 Homo sapiens lncRNA	Nucleus	Cytoplasm, Cytosol	Cytoplasm	Cytoplasm, Cytosol	Nucleus	Nucleus	Nucleus
52	>ID|30047|gene_id|BC030004|transcript_id|BC030004 BC030004 Mus musculus lncRNA	Nucleus	Nucleolus, Nucleus, Nucleoplasm	Nucleus	Nucleolus, Nucleus, Nucleoplasm	Nucleus/Exosome	Nucleus	Nucleus
53	>ID|56020|gene_id|ENSG00000224790|transcript_id|ENST00000430068 AP000704.5 Homo sapiens lncRNA	Cytosol	Ribosome	Cytoplasm	Ribosome	Nucleus/Cytosol	Nucleus	Nucleus
54	>ID|56321|gene_id|ENSG00000254002|transcript_id|ENST00000520375 RP11-213G6.2 Homo sapiens lncRNA	Cytosol	Cytoplasm, Cytosol	Cytoplasm	Cytoplasm, Cytosol	Nucleus/Exosome/ Cytosol/Membrane	Cytoplasm	Cytoplasm
55	>NCBI:100129617|BCALM(AC099524.1)|LOC100129617|Homo sapiens lncRNA	Nucleus	Cytoplasm, Cytosol	Cytoplasm	Cytoplasm, Cytosol	Nucleus	Cytoplasm	Cytoplasm
56	>ID|15575|gene_id|440823|transcript_id|NR_003491 MIAT Homo sapiens lncRNA	Nucleus	Nucleolus, Nucleus, Nucleoplasm	Nucleus	Nucleolus, Nucleus, Nucleoplasm	Nucleus/Exosome/ Membrane	Nucleus	Nucleus
57	>NCBI:400619|LINC00511 |LINC00511|Homo sapiens lncRNA	Ribosome	Ribosome	Ribosome	Ribosome	Nucleus/Exosome	Nucleus	Nucleus
58	>NCBI:101928752|LINC01043|LINC01043|Homo sapiens lncRNA	Cytoplasm	Nucleolus, Nucleus, Nucleoplasm	Nucleus	Nucleolus, Nucleus, Nucleoplasm	Nucleus/Exosome	Nucleus	Nucleus
59	>NCBI:388815|MIR99AHG|MIR99AHG|Homo sapiens lncRNA	Cytoplasm	Cytoplasm, Cytosol	Cytoplasm	Cytoplasm, Cytosol	Nucleus/Exosome	Nucleus	Nucleus
60	>NCBI:100873983|MAGI1-AS1|MAGI1-AS1|Homo sapiens lncRNA	Cytoplasm	Cytoplasm, Cytosol	Cytoplasm	Exosome	Nucleus	Nucleus	Nucleus
61	>ID|831|gene_id|102632457|transcript_id|XR_378115 Gm30524 Mus musculus lncRNA	Nucleus	Nucleolus, Nucleus, Nucleoplasm	Nucleus	Nucleolus, Nucleus, Nucleoplasm	Nucleus	Nucleus	Nucleus
62	>NCBI:100873947|LMLN-AS1|LMLN-AS1|Homo sapiens lncRNA	Cytoplasm	Cytoplasm, Cytosol	Cytoplasm	Cytoplasm, Cytosol	Nucleus	Nucleus	Nucleus
63	>ID|55963|gene_id|ENSG00000231993|transcript_id|ENST00000420537 RP1-85F18.5 Homo sapiens lncRNA	Ribosome	Ribosome	Cytoplasm	Cytoplasm, Cytosol	Nucleus/Exosome/ Cytosol/Membrane	Nucleus	Nucleus
64	>ID|23153|gene_id|68763|transcript_id|NR_027943 1110038B12Rik Mus musculus lncRNA	Nucleus	Nucleolus, Nucleus, Nucleoplasm	Nucleus	Nucleolus, Nucleus, Nucleoplasm	Nucleus	Nucleus	Nucleus
65	>ID|29791|gene_id|AK032810|transcript_id|AK032810 AK032810 Mus musculus lncRNA	Cytoplasm	Cytoplasm, Cytosol	Cytoplasm	Cytoplasm, Cytosol	Nucleus/Exosome	Cytoplasm	Cytoplasm
66	>ID|13631|gene_id|378805|transcript_id|NR_015431 LINC-PINT Homo sapiens lncRNA	Nucleus	Nucleolus, Nucleus, Nucleoplasm	Nucleus	Nucleolus, Nucleus, Nucleoplasm	Nucleus/Exosome	Nucleus	Nucleus
67	>NCBI:25845|PP7080|PP7080|Homo sapiens lncRNA	Cytosol	Nucleolus, Nucleus, Nucleoplasm	Cytoplasm	Nucleolus, Nucleus, Nucleoplasm	Nucleus/Exosome	Nucleus	Nucleus
68	>ID|29862|gene_id|AK081105|transcript_id|AK081105 AK081105 Mus musculus lncRNA	Nucleus	Nucleolus, Nucleus, Nucleoplasm	Cytoplasm	Nucleolus, Nucleus, Nucleoplasm	Nucleus	Cytoplasm	Nucleus
69	>ID|23029|gene_id|68246|transcript_id|NR_077218 1700112J05Rik Mus musculus lncRNA	Cytoplasm	Cytoplasm, Cytosol	Cytoplasm	Cytoplasm, Cytosol	Nucleus	Nucleus	Cytoplasm
70	>ID|56192|gene_id|ENSG00000229127|transcript_id|ENST00000452057 AC007038.7 Homo sapiens lncRNA	Cytoplasm	Cytoplasm, Cytosol	Cytoplasm	Cytoplasm, Cytosol	Nucleus/Cytosol	Cytoplasm	Nucleus
71	>ID|524|gene_id|101056138|transcript_id|XM_011238012 Gm20619 Mus musculus lncRNA	Nucleus	Nucleolus, Nucleus, Nucleoplasm	Cytoplasm	Nucleolus, Nucleus, Nucleoplasm	Nucleus	Nucleus	Nucleus
72	>ID|953|gene_id|102638523|transcript_id|XR_3	Cytoplasm	Cytoplasm,	Cytoplasm	Cytoplasm,	Nucleus/Exosome	Cytoplasm	Cytoplasm

	76167 Gm35066 Mus musculus lncRNA		Cytosol		Cytosol			
73	>ID|29876|gene_id|AK085783|transcript_id|AK085783 AK085783 Mus musculus lncRNA	Nucleus	Nucleolus, Nucleus, Nucleoplasm	Cytoplasm	Nucleolus, Nucleus, Nucleoplasm	Nucleus	Cytoplasm	Nucleus
74	>NCBI:100506622|LINC00540|LINC00540|Homo sapiens lncRNA	Cytoplasm	Cytoplasm, Cytosol	Ribosome	Cytoplasm, Cytosol	Nucleus/Exosome	Nucleus	Nucleus
75	>ID|29853|gene_id|AK078296|transcript_id|AK078296 AK078296 Mus musculus lncRNA	Cytoplasm	Cytoplasm, Cytosol	Cytoplasm	Cytoplasm, Cytosol	Nucleus	Nucleus	Cytoplasm
76	>ID|29952|gene_id|AK157498|transcript_id|AK157498 AK157498 Mus musculus lncRNA	Cytoplasm	Cytoplasm, Cytosol	Cytoplasm	Cytoplasm, Cytosol	Nucleus	Nucleus	Cytoplasm
77	>ID|22644|gene_id|67345|transcript_id|NM_026101 Herc4 Mus musculus lncRNA	Nucleus	Nucleolus, Nucleus, Nucleoplasm	Nucleus	Nucleolus, Nucleus, Nucleoplasm	Nucleus/Exosome/Membrane	Nucleus	Nucleus
78	>ID|56127|gene_id|ENSG00000234245|transcript_id|ENST00000428782 RP11-94I2.4 Homo sapiens lncRNA	Cytoplasm	Cytoplasm, Cytosol	Cytoplasm	Cytoplasm, Cytosol	Nucleus/Exosome/Membrane	Nucleus	Nucleus
79	>ID|29821|gene_id|AK044844|transcript_id|AK044844 AK044844 Mus musculus lncRNA	Cytoplasm	Cytoplasm, Cytosol	Cytoplasm	Cytoplasm, Cytosol	Nucleus	Cytoplasm	Cytoplasm
80	>NCBI:101927481|RP11-41O4.1|LINC01909|Homo sapiens lncRNA	Cytoplasm	Cytoplasm, Cytosol	Cytoplasm	Cytoplasm, Cytosol	Nucleus	Cytoplasm	Nucleus
81	>NCBI:100287616|LOXL1-AS1|LOXL1-AS1|Homo sapiens lncRNA	Cytosol	Nucleolus, Nucleus, Nucleoplasm	Cytoplasm	Nucleolus, Nucleus, Nucleoplasm	Nucleus/Exosome/Membrane	Nucleus	Nucleus
82	>ID|29802|gene_id|AK039415|transcript_id|AK039415 AK039415 Mus musculus lncRNA	Cytoplasm	Cytoplasm, Cytosol	Cytoplasm	Cytoplasm, Cytosol	Nucleus	Nucleus	Cytoplasm
83	>ID|56600|gene_id|ENSG00000258745|transcript_id|ENST00000557343 RP11-218E20.5 Homo sapiens lncRNA	Ribosome	Cytoplasm, Cytosol	Ribosome	Cytoplasm, Cytosol	Nucleus/Cytosol	Nucleus	Nucleus
84	>ID|56523|gene_id|ENSG00000258515|transcript_id|ENST00000555435 RP11-203M5.7 Homo sapiens lncRNA	Nucleus	Ribosome	Cytoplasm	Ribosome	Nucleus/Cytosol	Nucleus	Nucleus
85	>NCBI:285987|DLX6-AS1|DLX6-AS1|Homo sapiens lncRNA	Cytoplasm	Cytoplasm, Cytosol	Cytoplasm	Cytoplasm, Cytosol	Nucleus	Nucleus	Cytoplasm
86	>ID|23208|gene_id|68949|transcript_id|NM_001081005 Zfos1 Mus musculus lncRNA	Nucleus	Nucleolus, Nucleus, Nucleoplasm	Nucleus	Nucleolus, Nucleus, Nucleoplasm	Nucleus/Exosome	Nucleus	Nucleus
87	>ID|21936|gene_id|654809|transcript_id|NR_045871 4930428O21Rik Mus musculus lncRNA	Cytoplasm	Cytoplasm, Cytosol	Cytoplasm	Cytoplasm, Cytosol	Nucleus	Nucleus	Cytoplasm
88	>ID|10688|gene_id|285194|transcript_id|NR_015391 TUSC7 Homo sapiens lncRNA	Cytoplasm	Cytoplasm, Cytosol	Cytoplasm	Cytoplasm, Cytosol	Nucleus	Cytoplasm	Cytoplasm
89	>ID|29822|gene_id|AK045229|transcript_id|AK045229 AK045229 Mus musculus lncRNA	Cytoplasm	Cytoplasm, Cytosol	Cytoplasm	Cytoplasm, Cytosol	Nucleus	Cytoplasm	Cytoplasm
90	>NCBI:100507398|INTS6-AS1|INTS6-AS1|Homo sapiens lncRNA	Nucleus	Nucleolus, Nucleus, Nucleoplasm	Nucleus	Cytoplasm, Cytosol	Nucleus/Membrane	Nucleus	Nucleus
91	>ID|29867|gene_id|AK084036|transcript_id|AK084036 AK084036 Mus musculus lncRNA	Cytoplasm	Cytoplasm, Cytosol	Cytoplasm	Cytoplasm, Cytosol	Nucleus/Exosome	Cytoplasm	Cytoplasm
92	>ID|56308|gene_id|ENSG00000231125|transcript_id|ENST00000457162 AF129075.5 Homo sapiens lncRNA	Cytoplasm	Cytoplasm, Cytosol	Cytoplasm	Cytoplasm, Cytosol	Nucleus/Cytosol	Cytoplasm	Nucleus
93	>NCBI:100303728|SLC25A5-AS1|SLC25A5-AS1|Homo sapiens lncRNA	Cytosol	Cytoplasm, Cytosol	Cytoplasm	Cytoplasm, Cytosol	Nucleus/Exosome/Membrane	Nucleus	Nucleus
94	>ID|56004|gene_id|ENSG00000235245|transcript_id|ENST00000424450 RP11-122K13.12 Homo sapiens lncRNA	Exosome	Ribosome	Cytoplasm	Cytoplasm, Cytosol	Nucleus	Nucleus	Nucleus
95	>ID|21194|gene_id|622124|transcript_id|NR_040555 Gm20750 Mus musculus lncRNA	Cytoplasm	Cytoplasm, Cytosol	Cytoplasm	Cytoplasm, Cytosol	Nucleus	Cytoplasm	Cytoplasm
96	>ID|25238|gene_id|781|transcript_id|NM_000722 CACNA2D1 Homo sapiens lncRNA	Nucleus	Nucleolus, Nucleus, Nucleoplasm	Nucleus	Nucleolus, Nucleus, Nucleoplasm	Nucleus/Exosome/Membrane	Nucleus	Nucleus
97	>ID|56658|gene_id|ENSG00000254519|transcript_id|ENST00000529769 CTD-2210P24.1 Homo sapiens lncRNA	Cytosol	Cytoplasm, Cytosol	Nucleus	Nucleolus, Nucleus, Nucleoplasm	Nucleus/Exosome/Cytosol	Cytoplasm	Cytoplasm

Supplementary Table S4. The detailed prediction results of iLoc-miRNA, DeepLocRNA, and RNALoc-LM on the miRNA independent test set.

No.	miRNA	iLoc-miRNA	DeepLocRNA	RNALoc-LM	True Labels
1	>0,1,Gene_id:MI0000292,Gene_name:hsa-mir-216a,Gene_symbol:hsa-mir-216a,Origin_loc:Cytoplasm|1	Intracellular	Nucleus/Cytosol/Cytoplasm	Intracellular	Intracellular
2	>0,1,Gene_id:MI0006355,Gene_name:hsa-mir-1293,Gene_symbol:hsa-mir-1293,Origin_loc:Cytoplasm|1	Intracellular	Nucleus/Cytosol/Cytoplasm	Intracellular	Intracellular
3	>1,0,Gene_id:MIMAT0015075,Gene_name:hsa-miR-3191,Gene_symbol:hsa-miR-3191-3p,Origin_loc:Microvesicle|0	Extracellular	Exosome/Microvesicle	Extracellular	Extracellular
4	>1,0,Gene_id:MIMAT0000268,Gene_name:hsa-miR-211-5p,Gene_symbol:hsa-miR-211-5p,Origin_loc:Exosome|0	Extracellular	Exosome/Microvesicle	Extracellular	Extracellular
5	>1,0,Gene_id:MIMAT0002187,Gene_name:kshv-miR-K12-7,Gene_symbol:kshv-miR-K12-7-3p,Origin_loc:Exosome|0	Extracellular	Exosome/Microvesicle	Extracellular	Extracellular
6	>1,0,Gene_id:MIMAT0002849,Gene_name:hsa-miR-524-5p,Gene_symbol:hsa-miR-524-5p,Origin_loc:Exosome|0	Extracellular	Exosome/Microvesicle	Extracellular	Extracellular
7	>1,0,Gene_id:MIMAT0019840,Gene_name:hsa-miR-451b,Gene_symbol:hsa-miR-451b,Origin_loc:Exosome|0	Extracellular	Exosome/Microvesicle	Extracellular	Extracellular
8	>0,1,Gene_id:MI0015898,Gene_name:hsa-mir-4289,Gene_symbol:hsa-mir-4289,Origin_loc:Nucleus|1	Intracellular	Nucleus/Cytosol/Cytoplasm	Intracellular	Intracellular
9	>1,0,Gene_id:MI0007261,Gene_name:hsa-mir-103b-1,Gene_symbol:hsa-mir-103b-1,Origin_loc:Exosome|0	Both	Nucleus/Cytosol/Cytoplasm	Intracellular	Extracellular
10	>1,0,Gene_id:MIMAT0003235,Gene_name:hsa-miR-570-3p,Gene_symbol:hsa-miR-570-3p,Origin_loc:Exosome|0	Extracellular	Exosome/Microvesicle	Extracellular	Extracellular
11	>1,0,Gene_id:MIMAT0019708,Gene_name:hsa-miR-4646-3p,Gene_symbol:hsa-miR-4646-3p,Origin_loc:Exosome|0	Extracellular	Exosome/Microvesicle	Extracellular	Extracellular
12	>1,0,Gene_id:MIMAT0004803,Gene_name:hsa-miR-548a-5p,Gene_symbol:hsa-miR-548a-5p,Origin_loc:Exosome|0	Extracellular	Exosome/Microvesicle	Extracellular	Extracellular
13	>0,1,Gene_id:MI0006327,Gene_name:hsa-mir-1237,Gene_symbol:hsa-mir-1237,Origin_loc:Nucleus|1	Intracellular	Nucleus/Cytosol/Cytoplasm	Intracellular	Intracellular
14	>1,0,Gene_id:MIMAT0003307,Gene_name:hsa-miR-637,Gene_symbol:hsa-miR-637,Origin_loc:Exosome|0	Extracellular	Exosome/Microvesicle	Extracellular	Extracellular
15	>0,1,Gene_id:MI0006356,Gene_name:hsa-mir-1294,Gene_symbol:hsa-mir-1294,Origin_loc:Nucleus|1	Intracellular	Nucleus/Cytosol/Cytoplasm	Intracellular	Intracellular
16	>1,0,Gene_id:MI0003142,Gene_name:hsa-mir-498,Gene_symbol:hsa-mir-498,Origin_loc:Exosome|0	Intracellular	Nucleus/Cytosol/Cytoplasm	Intracellular	Extracellular
17	>1,0,Gene_id:MIMAT0025856,Gene_name:hsa-miR-6724-5p,Gene_symbol:hsa-miR-6724-5p,Origin_loc:Exosome|0	Extracellular	Exosome/Microvesicle	Extracellular	Extracellular
18	>0,1,Gene_id:MI0003583,Gene_name:hsa-mir-576,Gene_symbol:hsa-mir-576,Origin_loc:Cytoplasm|1	Intracellular	Nucleus/Cytosol/Cytoplasm	Intracellular	Intracellular
19	>1,0,Gene_id:MIMAT0015022,Gene_name:hsa-miR-3149,Gene_symbol:hsa-miR-3149,Origin_loc:Microvesicle|0	Extracellular	Exosome/Microvesicle	Extracellular	Extracellular
20	>1,0,Gene_id:MIMAT0003881,Gene_name:hsa-miR-668,Gene_symbol:hsa-miR-668-3p,Origin_loc:Exosome|0	Extracellular	Exosome/Microvesicle	Extracellular	Extracellular
21	>1,0,Gene_id:MIMAT0018984,Gene_name:hsa-miR-378h,Gene_symbol:hsa-miR-378h,Origin_loc:Exosome|0	Extracellular	Exosome/Microvesicle	Extracellular	Extracellular
22	>1,0,Gene_id:MIMAT0022929,Gene_name:hsa-miR-758-5p,Gene_symbol:hsa-miR-758-5p,Origin_loc:Exosome|0	Extracellular	Exosome/Microvesicle	Extracellular	Extracellular
23	>0,1,Gene_id:MI0006371,Gene_name:hsa-mir-1304,Gene_symbol:hsa-mir-1304,Origin_loc:Cytoplasm|1	Intracellular	Nucleus/Cytosol/Cytoplasm	Intracellular	Intracellular
24	>1,0,Gene_id:MIMAT0005582,Gene_name:hsa-miR-1228-5p,Gene_symbol:hsa-miR-1228-5p,Origin_loc:Exosome|0	Extracellular	Exosome/Microvesicle	Extracellular	Extracellular
25	>1,0,Gene_id:MIMAT0004589,Gene_name:hsa-miR-30b-3p,Gene_symbol:hsa-miR-30b-3p,Origin_loc:Exosome|0	Extracellular	Exosome/Microvesicle	Extracellular	Extracellular
26	>1,0,Gene_id:MIMAT0000723,Gene_name:hsa-miR-371,Gene_symbol:hsa-miR-371a-3p,Origin_loc:Exosome|0	Extracellular	Exosome/Microvesicle	Extracellular	Extracellular
27	>1,0,Gene_id:MIMAT0003712,Gene_name:kshv-miR-K12-12,Gene_symbol:kshv-miR-K12-12-5p,Origin_loc:Exosome|0	Extracellular	Exosome/Microvesicle	Extracellular	Extracellular
28	>1,0,Gene_id:MIMAT0007883,Gene_name:hsa-miR-1909,Gene_symbol:hsa-miR-1909-3p,Origin_loc:Exosome|0	Extracellular	Exosome/Microvesicle	Extracellular	Extracellular
29	>1,0,Gene_id:MIMAT0005907,Gene_name:hsa-miR-1256,Gene_symbol:hsa-miR-1256,Origin_loc:Exosome|0	Extracellular	Exosome/Microvesicle	Extracellular	Extracellular
30	>0,1,Gene_id:MI0005523,Gene_name:hsa-mir-298,Gene_symbol:hsa-mir-298,Origin_loc:Nucleus|1	Intracellular	Nucleus/Cytosol/Cytoplasm	Intracellular	Intracellular
31	>1,0,Gene_id:MIMAT0007885,Gene_name:hsa-miR-1911-5p,Gene_symbol:hsa-miR-1911-5p,Origin_loc:Exosome|0	Extracellular	Exosome/Microvesicle	Extracellular	Extracellular
32	>1,0,Gene_id:MIMAT0003386,Gene_name:hsa-miR-376a-5p,Gene_symbol:hsa-miR-376a-5p,Origin_loc:Exosome|0	Extracellular	Exosome/Microvesicle	Extracellular	Extracellular
33	>1,0,Gene_id:MIMAT0015027,Gene_name:hsa-miR-3074-3p,Gene_symbol:hsa-miR-3074-3p,Origin_loc:Exosome|0	Extracellular	Exosome/Microvesicle	Extracellular	Extracellular
34	>1,0,Gene_id:MIMAT0022692,Gene_name:hsa-miR-181b-3p,Gene_symbol:hsa-miR-181b-3p,Origin_loc:Exosome|0	Extracellular	Exosome/Microvesicle	Extracellular	Extracellular
35	>0,1,Gene_id:MI0000764,Gene_name:hsa-mir-363,Gene_symbol:hsa-mir-363,Origin_loc:Nucleus|1	Intracellular	Nucleus/Cytosol/Cytoplasm	Intracellular	Intracellular
36	>1,0,Gene_id:MIMAT0031011,Gene_name:hsa-miR-8084,Gene_symbol:hsa-miR-8084,Origin_loc:Exosome|0	Extracellular	Exosome/Microvesicle	Extracellular	Extracellular
37	>1,0,Gene_id:MI0000479,Gene_name:hsa-mir-150,Gene_symbol:hsa-mir-150,Origin_loc:Microvesicle|0	Intracellular	Nucleus/Cytosol/Cytoplasm	Intracellular	Extracellular
38	>1,0,Gene_id:MIMAT0000251,Gene_name:hsa-miR-147,Gene_symbol:hsa-miR-147a,Origin_loc:Exosome|0	Extracellular	Exosome/Microvesicle	Extracellular	Extracellular
39	>0,1,Gene_id:MI0003941,Gene_name:hsa-mir-761,Gene_symbol:hsa-mir-761,Origin_loc:Nucleus|1	Extracellular	Nucleus/Cytosol/Cytoplasm/Microvesicle	Intracellular	Intracellular
40	>1,0,Gene_id:MIMAT0019055,Gene_name:hsa-miR-4518,Gene_symbol:hsa-miR-4518,Origin_loc:Exosome|0	Extracellular	Exosome/Microvesicle	Extracellular	Extracellular
41	>0,1,Gene_id:MI0000783,Gene_name:hsa-mir-375,Gene_symbol:hsa-mir-375,Origin_loc:Cytoplasm|1	Both	Nucleus/Cytosol/Cytoplasm	Intracellular	Intracellular
42	>1,0,Gene_id:MIMAT0027359,Gene_name:hsa-miR-6729-5p,Gene_symbol:hsa-miR-6729-5p,Origin_loc:Exosome|0	Extracellular	Exosome/Microvesicle	Extracellular	Extracellular
43	>1,0,Gene_id:MIMAT0010195,Gene_name:hsa-let-7a-2-3p,Gene_symbol:hsa-let-7a-2-3p,Origin_loc:Exosome|0	Extracellular	Exosome/Microvesicle	Extracellular	Extracellular
44	>0,1,Gene_id:MI0014157,Gene_name:hsa-mir-466,Gene_symbol:hsa-mir-466,Origin_loc:Nucleus|1	Intracellular	Nucleus/Cytosol/Cytoplasm	Intracellular	Intracellular

45	>1,0,Gene_id:MIMAT0004586,Gene_name:hsa-miR-15b*,Gene_symbol:hsa-miR-15b-3p,Origin_loc:Exosome|0	Extracellular	Exosome/Microvesicle	Extracellular	Extracellular
46	>1,0,Gene_id:MIMAT0005878,Gene_name:hsa-miR-1287,Gene_symbol:hsa-miR-1287-5p,Origin_loc:Exosome|0	Extracellular	Exosome/Microvesicle	Extracellular	Extracellular
47	>1,0,Gene_id:MIMAT0022711,Gene_name:hsa-miR-660-3p,Gene_symbol:hsa-miR-660-3p,Origin_loc:Exosome|0	Extracellular	Exosome/Microvesicle	Extracellular	Extracellular
48	>1,0,Gene_id:MIMAT0007890,Gene_name:hsa-miR-1914-star,Gene_symbol:hsa-miR-1914-3p,Origin_loc:Microvesicle|0	Extracellular	Exosome/Microvesicle	Extracellular	Extracellular
49	>1,0,Gene_id:MIMAT0003318,Gene_name:hsa-miR-648,Gene_symbol:hsa-miR-648,Origin_loc:Exosome|0	Extracellular	Exosome/Microvesicle	Extracellular	Extracellular
50	>1,0,Gene_id:MIMAT0002818,Gene_name:hsa-miR-496,Gene_symbol:hsa-miR-496,Origin_loc:Exosome|0	Extracellular	Exosome/Microvesicle	Extracellular	Extracellular
51	>1,0,Gene_id:MIMAT0022838,Gene_name:hsa-miR-1185-1-3p,Gene_symbol:hsa-miR-1185-1-3p,Origin_loc:Exosome|0	Extracellular	Exosome/Microvesicle	Extracellular	Extracellular
52	>0,1,Gene_id:MI0006430,Gene_name:hsa-mir-1283-2,Gene_symbol:hsa-mir-1283-2,Origin_loc:Nucleus|1	Intracellular	Nucleus/Cytosol/Cytoplasm	Intracellular	Intracellular
53	>1,0,Gene_id:MIMAT0016879,Gene_name:hsa-miR-4258,Gene_symbol:hsa-miR-4258,Origin_loc:Exosome|0	Extracellular	Exosome/Microvesicle	Extracellular	Extracellular
54	>0,1,Gene_id:MIMAT0022833,Gene_name:hsa-miR-365b-5p,Gene_symbol:hsa-miR-365b-5p,Origin_loc:Mitochondrion|1	Extracellular	Exosome/Microvesicle	Extracellular	Intracellular
55	>0,1,Gene_id:MI0005768,Gene_name:hsa-mir-943,Gene_symbol:hsa-mir-943,Origin_loc:Nucleus|1	Intracellular	Nucleus/Cytosol/Cytoplasm	Intracellular	Intracellular
56	>0,1,Gene_id:MI0009983,Gene_name:hsa-mir-1973,Gene_symbol:hsa-mir-1973,Origin_loc:Nucleus|1	Extracellular	Nucleus/Exosome/Cytosol/ Cytoplasm/Microvesicle	Extracellular	Intracellular
57	>1,0,Gene_id:MI0007262,Gene_name:hsa-mir-103b-2,Gene_symbol:hsa-mir-103b-2,Origin_loc:Exosome|0	Both	Nucleus/Cytosol/Cytoplasm /Microvesicle	Intracellular	Extracellular
58	>1,0,Gene_id:MI0006431,Gene_name:hsa-mir-1284,Gene_symbol:hsa-mir-1284,Origin_loc:Exosome|0	Intracellular	Nucleus/Exosome/Cytosol/ Cytoplasm/Microvesicle	Intracellular	Extracellular
59	>1,0,Gene_id:MIMAT0004920,Gene_name:hsa-miR-541-3p,Gene_symbol:hsa-miR-541-3p,Origin_loc:Exosome|0	Extracellular	Exosome/Microvesicle	Extracellular	Extracellular
60	>0,1,Gene_id:MI0006358,Gene_name:hsa-mir-1297,Gene_symbol:hsa-mir-1297,Origin_loc:Nucleus|1	Intracellular	Nucleus/Cytosol/Cytoplasm	Intracellular	Intracellular
61	>1,0,Gene_id:MI0006352,Gene_name:hsa-mir-1290,Gene_symbol:hsa-mir-1290,Origin_loc:Exosome|0	Intracellular	Nucleus/Cytosol/Cytoplasm /Microvesicle	Intracellular	Extracellular
62	>1,0,Gene_id:MIMAT0019976,Gene_name:hsa-miR-4799-5p,Gene_symbol:hsa-miR-4799-5p,Origin_loc:Exosome|0	Extracellular	Exosome/Microvesicle	Extracellular	Extracellular
63	>0,1,Gene_id:MI0000769,Gene_name:hsa-mir-365-2,Gene_symbol:hsa-mir-365b,Origin_loc:Cytoplasm|1	Intracellular	Nucleus/Cytosol/Cytoplasm	Intracellular	Intracellular
64	>0,1,Gene_id:MI0014228,Gene_name:hsa-mir-3065,Gene_symbol:hsa-mir-3065,Origin_loc:Cytoplasm|1	Intracellular	Nucleus/Cytosol/Cytoplasm	Intracellular	Intracellular
65	>0,1,Gene_id:MI0003630,Gene_name:hsa-mir-548c,Gene_symbol:hsa-mir-548c,Origin_loc:Nucleus|1	Intracellular	Nucleus/Cytosol/Cytoplasm	Intracellular	Intracellular
66	>1,0,Gene_id:MIMAT0004911,Gene_name:hsa-miR-874,Gene_symbol:hsa-miR-874-3p,Origin_loc:Exosome|0	Extracellular	Exosome/Microvesicle	Extracellular	Extracellular
67	>0,1,Gene_id:MI0014188,Gene_name:hsa-mir-3159,Gene_symbol:hsa-mir-3159,Origin_loc:Nucleus|1	Intracellular	Nucleus/Cytosol/Cytoplasm	Intracellular	Intracellular
68	>0,1,Gene_id:MI0000811,Gene_name:hsa-mir-148b,Gene_symbol:hsa-mir-148b,Origin_loc:Cytoplasm|1	Intracellular	Nucleus/Cytosol/Cytoplasm	Intracellular	Intracellular
69	>0,1,Gene_id:MI0003587,Gene_name:hsa-mir-580,Gene_symbol:hsa-mir-580,Origin_loc:Cytoplasm|1	Intracellular	Nucleus/Cytosol/Cytoplasm /Microvesicle	Intracellular	Intracellular
70	>1,0,Gene_id:MIMAT0015002,Gene_name:hsa-miR-466,Gene_symbol:hsa-miR-466,Origin_loc:Exosome|0	Extracellular	Exosome/Microvesicle	Extracellular	Extracellular
71	>0,1,Gene_id:MI0006416,Gene_name:hsa-mir-1276,Gene_symbol:hsa-mir-1276,Origin_loc:Nucleus|1	Intracellular	Nucleus/Cytosol/Cytoplasm	Intracellular	Intracellular
72	>0,1,Gene_id:MI0003190,Gene_name:hsa-mir-505,Gene_symbol:hsa-mir-505,Origin_loc:Cytoplasm|1	Intracellular	Nucleus/Cytosol/Cytoplasm	Intracellular	Intracellular
73	>1,0,Gene_id:MIMAT0005459,Gene_name:hsa-miR-1224-3p,Gene_symbol:hsa-miR-1224-3p,Origin_loc:Exosome|0	Extracellular	Exosome/Microvesicle	Extracellular	Extracellular
74	>1,0,Gene_id:MI0006428,Gene_name:hsa-mir-1281,Gene_symbol:hsa-mir-1281,Origin_loc:Exosome|0	Extracellular	Nucleus/Cytosol/Cytoplasm /Microvesicle	Intracellular	Extracellular
75	>0,1,Gene_id:MI0003145,Gene_name:hsa-mir-519e,Gene_symbol:hsa-mir-519e,Origin_loc:Nucleus|1	Intracellular	Nucleus/Cytosol/Cytoplasm	Intracellular	Intracellular
76	>0,1,Gene_id:MI0005766,Gene_name:hsa-mir-941-4,Gene_symbol:hsa-mir-941-4,Origin_loc:Cytoplasm|1	Intracellular	Nucleus/Cytosol/Cytoplasm	Intracellular	Intracellular
77	>0,1,Gene_id:MI0000293,Gene_name:hsa-mir-217,Gene_symbol:hsa-mir-217,Origin_loc:Cytoplasm|1	Intracellular	Nucleus/Cytosol/Cytoplasm	Intracellular	Intracellular
78	>1,0,Gene_id:MIMAT0018446,Gene_name:hsa-miR-548z,Gene_symbol:hsa-miR-548z,Origin_loc:Exosome|0	Extracellular	Exosome/Microvesicle	Extracellular	Extracellular
79	>1,0,Gene_id:MIMAT0003331,Gene_name:hsa-miR-655,Gene_symbol:hsa-miR-655-3p,Origin_loc:Exosome|0	Extracellular	Exosome/Microvesicle	Extracellular	Extracellular
80	>1,0,Gene_id:MI0003786,Gene_name:hsa-mir-1323,Gene_symbol:hsa-mir-1323,Origin_loc:Exosome|0	Intracellular	Nucleus/Cytosol/Cytoplasm	Intracellular	Extracellular
81	>1,0,Gene_id:MIMAT0004501,Gene_name:hsa-miR-27a-5p,Gene_symbol:hsa-miR-27a-5p,Origin_loc:Exosome|0	Extracellular	Exosome/Microvesicle	Extracellular	Extracellular
82	>1,0,Gene_id:MIMAT0019932,Gene_name:hsa-miR-4776-5p,Gene_symbol:hsa-miR-4776-5p,Origin_loc:Exosome|0	Extracellular	Exosome/Microvesicle	Extracellular	Extracellular
83	>0,1,Gene_id:MI0014177,Gene_name:hsa-mir-3150,Gene_symbol:hsa-mir-3150a,Origin_loc:Nucleus|1	Intracellular	Nucleus/Cytosol/Cytoplasm	Intracellular	Intracellular
84	>1,0,Gene_id:MIMAT0002834,Gene_name:hsa-miR-520a-3p,Gene_symbol:hsa-miR-520a-3p,Origin_loc:Exosome|0	Extracellular	Exosome/Microvesicle	Extracellular	Extracellular
85	>1,0,Gene_id:MIMAT0003423,Gene_name:ebv-miR-BART12,Gene_symbol:ebv-miR-BART12,Origin_loc:Exosome|0	Extracellular	Exosome/Microvesicle	Extracellular	Extracellular
86	>1,0,Gene_id:MIMAT0003271,Gene_name:hsa-miR-603,Gene_symbol:hsa-miR-603,Origin_loc:Exosome|0	Extracellular	Exosome/Microvesicle	Extracellular	Extracellular
87	>1,0,Gene_id:MIMAT0003163,Gene_name:hsa-miR-539-5p,Gene_symbol:hsa-miR-539-5p,Origin_loc:Exosome|0	Extracellular	Exosome/Microvesicle	Extracellular	Extracellular
88	>0,1,Gene_id:MI0003657,Gene_name:hsa-mir-642,Gene_symbol:hsa-mir-642a,Origin_loc:Cytoplasm|1	Intracellular	Nucleus/Cytosol/Cytoplasm	Intracellular	Intracellular
89	>0,1,Gene_id:MI0015836,Gene_name:hsa-mir-4306,Gene_symbol:hsa-mir-4306,Origin_loc:Nucleus|1	Intracellular	Nucleus/Cytosol/Cytoplasm	Intracellular	Intracellular
90	>1,0,Gene_id:MIMAT0005589,Gene_name:hsa-miR-1234,Gene_symbol:hsa-miR-1234-3p,Origin_loc:Exosome|0	Extracellular	Exosome/Microvesicle	Extracellular	Extracellular
91	>1,0,Gene_id:MIMAT0004929,Gene_name:hsa-miR-190b,Gene_symbol:hsa-miR-190b-5p,Origin_loc:Exosome|0	Extracellular	Exosome/Microvesicle	Extracellular	Extracellular
92	>1,0,Gene_id:MIMAT0002829,Gene_name:hsa-miR-519e-3p,Gene_symbol:hsa-miR-519e-3p,Origin_loc:Exosome|0	Extracellular	Exosome/Microvesicle	Extracellular	Extracellular

<table>
<tr><td>93</td><td>>1,0,Gene_id:MIMAT0003257,Gene_name:hsa-miR-550*,Gene_symbol:hsa-miR-550a-3p,Origin_loc:Exosome|0</td><td>Extracellular</td><td>Exosome/Microvesicle</td><td>Extracellular</td><td>Extracellular</td></tr>
<tr><td>94</td><td>>1,0,Gene_id:MIMAT0015058,Gene_name:hsa-miR-3180-3p,Gene_symbol:hsa-miR-3180-3p,Origin_loc:Exosome|0</td><td>Extracellular</td><td>Exosome/Microvesicle</td><td>Extracellular</td><td>Extracellular</td></tr>
<tr><td>95</td><td>>1,0,Gene_id:MIMAT0005796,Gene_name:hsa-miR-1271-5p,Gene_symbol:hsa-miR-1271-5p,Origin_loc:Exosome|0</td><td>Extracellular</td><td>Exosome/Microvesicle</td><td>Extracellular</td><td>Extracellular</td></tr>
<tr><td>96</td><td>>0,1,Gene_id:MI0005545,Gene_name:hsa-mir-190b,Gene_symbol:hsa-mir-190b,Origin_loc:Nucleus|1</td><td>Intracellular</td><td>Nucleus/Cytosol/Cytoplasm</td><td>Intracellular</td><td>Intracellular</td></tr>
<tr><td>97</td><td>>1,0,Gene_id:MIMAT0000771,Gene_name:hsa-miR-325,Gene_symbol:hsa-miR-325,Origin_loc:Exosome|0</td><td>Extracellular</td><td>Exosome/Microvesicle</td><td>Extracellular</td><td>Extracellular</td></tr>
<tr><td>98</td><td>>1,0,Gene_id:MIMAT0016907,Gene_name:hsa-miR-4281,Gene_symbol:hsa-miR-4281,Origin_loc:Microvesicle|0</td><td>Extracellular</td><td>Exosome/Microvesicle</td><td>Extracellular</td><td>Extracellular</td></tr>
<tr><td>99</td><td>>0,1,Gene_id:MI0015974,Gene_name:hsa-mir-1244-2,Gene_symbol:hsa-mir-1244-2,Origin_loc:Cytoplasm|1</td><td>Intracellular</td><td>Nucleus/Cytosol/Cytoplasm</td><td>Intracellular</td><td>Intracellular</td></tr>
<tr><td>100</td><td>>1,0,Gene_id:MIMAT0027452,Gene_name:hsa-miR-6776-5p,Gene_symbol:hsa-miR-6776-5p,Origin_loc:Exosome|0</td><td>Extracellular</td><td>Exosome/Microvesicle</td><td>Extracellular</td><td>Extracellular</td></tr>
<tr><td>101</td><td>>1,0,Gene_id:MIMAT0004916,Gene_name:hsa-miR-888,Gene_symbol:hsa-miR-888-5p,Origin_loc:Exosome|0</td><td>Extracellular</td><td>Exosome/Microvesicle</td><td>Extracellular</td><td>Extracellular</td></tr>
<tr><td>102</td><td>>0,1,Gene_id:MIMAT0018925,Gene_name:hsa-miR-1268b,Gene_symbol:hsa-miR-1268b,Origin_loc:Mitochondrion|1</td><td>Extracellular</td><td>Exosome/Microvesicle</td><td>Extracellular</td><td>Intracellular</td></tr>
<tr><td>103</td><td>>0,1,Gene_id:MI0014145,Gene_name:hsa-mir-3128,Gene_symbol:hsa-mir-3128,Origin_loc:Nucleus|1</td><td>Intracellular</td><td>Nucleus/Cytosol/Cytoplasm</td><td>Intracellular</td><td>Intracellular</td></tr>
<tr><td>104</td><td>>0,1,Gene_id:MIMAT0005914,Gene_name:hsa-miR-1262,Gene_symbol:hsa-miR-1262,Origin_loc:Mitochondrion|1</td><td>Extracellular</td><td>Exosome/Microvesicle</td><td>Extracellular</td><td>Intracellular</td></tr>
<tr><td>105</td><td>>1,0,Gene_id:MIMAT0018927,Gene_name:hsa-miR-378e,Gene_symbol:hsa-miR-378e,Origin_loc:Exosome|0</td><td>Extracellular</td><td>Exosome/Microvesicle</td><td>Extracellular</td><td>Extracellular</td></tr>
<tr><td>106</td><td>>1,0,Gene_id:MIMAT0004907,Gene_name:hsa-miR-892a,Gene_symbol:hsa-miR-892a,Origin_loc:Microvesicle|0</td><td>Extracellular</td><td>Exosome/Microvesicle</td><td>Extracellular</td><td>Extracellular</td></tr>
<tr><td>107</td><td>>0,1,Gene_id:MI0003161,Gene_name:hsa-mir-517a,Gene_symbol:hsa-mir-517a,Origin_loc:Nucleus|1</td><td>Intracellular</td><td>Nucleus/Cytosol/Cytoplasm</td><td>Intracellular</td><td>Intracellular</td></tr>
<tr><td>108</td><td>>1,0,Gene_id:MIMAT0015035,Gene_name:hsa-miR-3161,Gene_symbol:hsa-miR-3161,Origin_loc:Exosome|0</td><td>Extracellular</td><td>Exosome/Microvesicle</td><td>Extracellular</td><td>Extracellular</td></tr>
<tr><td>109</td><td>>1,0,Gene_id:MI0015863,Gene_name:hsa-mir-4255,Gene_symbol:hsa-mir-4255,Origin_loc:Microvesicle|0</td><td>Intracellular</td><td>Nucleus/Cytosol/Cytoplasm</td><td>Intracellular</td><td>Extracellular</td></tr>
<tr><td>110</td><td>>1,0,Gene_id:MIMAT0016873,Gene_name:hsa-miR-4322,Gene_symbol:hsa-miR-4322,Origin_loc:Microvesicle|0</td><td>Extracellular</td><td>Exosome/Microvesicle</td><td>Extracellular</td><td>Extracellular</td></tr>
<tr><td>111</td><td>>1,0,Gene_id:MIMAT0003221,Gene_name:hsa-miR-557,Gene_symbol:hsa-miR-557,Origin_loc:Exosome|0</td><td>Extracellular</td><td>Exosome/Microvesicle</td><td>Extracellular</td><td>Extracellular</td></tr>
<tr><td>112</td><td>>0,1,Gene_id:MI0003577,Gene_name:hsa-mir-570,Gene_symbol:hsa-mir-570,Origin_loc:Nucleus|1</td><td>Intracellular</td><td>Nucleus/Cytosol/Cytoplasm</td><td>Intracellular</td><td>Intracellular</td></tr>
<tr><td>113</td><td>>1,0,Gene_id:MIMAT0019886,Gene_name:hsa-miR-4749-3p,Gene_symbol:hsa-miR-4749-3p,Origin_loc:Exosome|0</td><td>Extracellular</td><td>Exosome/Microvesicle</td><td>Extracellular</td><td>Extracellular</td></tr>
</table>

Supplementary Table S5. The detailed prediction results of RNALight, CellCircLoc, and RNALoc-LM on the circRNA independent test set.

<table>
<tr><th>No.</th><th>circRNA</th><th>RNALight</th><th>CellCircLoc</th><th>RNALoc-LM</th><th>True Labels</th></tr>
<tr><td>1</td><td>>circRNA:chr20:63928334,63931734|circbase_id:---|type:normal,cancer|position:Exon,Intron|host_gene:DNAJC5</td><td>Cytoplasm</td><td>Cytoplasm</td><td>Cytoplasm</td><td>Cytoplasm</td></tr>
<tr><td>2</td><td>>circRNA:chr16:67951284,67951886|circbase_id:---|type:cancer|position:Exon,Intron|host_gene:SLC12A4</td><td>Cytoplasm</td><td>Nucleus</td><td>Nucleus</td><td>Cytoplasm</td></tr>
<tr><td>3</td><td>>circRNA:chr22:36476732,36477526|circbase_id:hsa_circ_0116523|type:normal,cancer| position:Exon,Intron|host_gene:TXN2</td><td>Cytoplasm</td><td>Nucleus</td><td>Cytoplasm</td><td>Nucleus</td></tr>
<tr><td>4</td><td>>circRNA:chr6:128476362,128476904|circbase_id:---|type:cancer|position:Intron|host_gene:PTPRK</td><td>Nucleus</td><td>Nucleus</td><td>Nucleus</td><td>Nucleus</td></tr>
<tr><td>5</td><td>>circRNA:chr1:156232319,156233728|circbase_id:hsa_circ_0014684|type: normal,cance|position:Exon,Intron|host_gene:PMF1-BGLAP</td><td>Cytoplasm</td><td>Nucleus</td><td>Cytoplasm</td><td>Nucleus</td></tr>
<tr><td>6</td><td>>circRNA:chr3:11826717,11833151|circbase_id:---|type:normal,cancer|position:Exon,Intron|host_gene:TAMM41</td><td>Nucleus</td><td>Cytoplasm</td><td>Cytoplasm</td><td>Cytoplasm</td></tr>
<tr><td>7</td><td>>circRNA:chr22:29284410,29284504|circbase_id:---|type:cancer|position:Intron|host_gene:EWSR1</td><td>Nucleus</td><td>Nucleus</td><td>Nucleus</td><td>Nucleus</td></tr>
<tr><td>8</td><td>>circRNA:chr6:131830106,131830199|circbase_id:---|type:cancer|position:Intron|host_gene:ENPP1</td><td>Nucleus</td><td>Nucleus</td><td>Nucleus</td><td>Nucleus</td></tr>
<tr><td>9</td><td>>circRNA:chr17:59565657,59566668|circbase_id:---|type:normal,cancer|position:Exon,Intron|host_gene:DHX40</td><td>Cytoplasm</td><td>Nucleus</td><td>Nucleus</td><td>Nucleus</td></tr>
<tr><td>10</td><td>>circRNA:chr10:32953437,32954013|circbase_id:---|type:cancer|position:Exon,Intron|host_gene:ITGB1</td><td>Nucleus</td><td>Nucleus</td><td>Nucleus</td><td>Nucleus</td></tr>
<tr><td>11</td><td>>circRNA:chr17:40956336,40956569|circbase_id:---|type:cancer|position:Intron|host_gene:AC004231.1</td><td>Nucleus</td><td>Nucleus</td><td>Nucleus</td><td>Nucleus</td></tr>
<tr><td>12</td><td>>circRNA:chr2:138013319,138013428|circbase_id:---|type:cancer|position:Intron|host_gene:HNMT</td><td>Nucleus</td><td>Nucleus</td><td>Nucleus</td><td>Nucleus</td></tr>
<tr><td>13</td><td>>circRNA:chr9:107300140,107322118|circbase_id:hsa_circ_0087856|type:normal,cancer|position:Exon,Intron|host_gene:RAD23B</td><td>Cytoplasm</td><td>Cytoplasm</td><td>Cytoplasm</td><td>Cytoplasm</td></tr>
<tr><td>14</td><td>>circRNA:chr1:212835872,212836187|circbase_id:hsa_circ_0111904|type:normal,cancer|position:Exon,Intron|host_gene:SPATA45</td><td>Cytoplasm</td><td>Cytoplasm</td><td>Cytoplasm</td><td>Cytoplasm</td></tr>
<tr><td>15</td><td>>circRNA:chr10:34547642,34547885|circbase_id:---|type:cancer|position:Intron|host_gene:PARD3</td><td>Nucleus</td><td>Nucleus</td><td>Nucleus</td><td>Nucleus</td></tr>
<tr><td>16</td><td>>circRNA:chr3:48630437,48632396|circbase_id:---|type:normal,cancer|position:Exon,Intron|host_gene:SLC26A6</td><td>Nucleus</td><td>Cytoplasm</td><td>Cytoplasm</td><td>Nucleus</td></tr>
<tr><td>17</td><td>>circRNA:chr7:6641824,6644593|circbase_id:---|type:cancer|position:Exon,Intron|host_gene:ZNF316</td><td>Nucleus</td><td>Cytoplasm</td><td>Cytoplasm</td><td>Cytoplasm</td></tr>
<tr><td>18</td><td>>circRNA:chr1:155253642,155253729|circbase_id:---|type:cancer|position:Exon|host_gene:FAM189B</td><td>Nucleus</td><td>Nucleus</td><td>Nucleus</td><td>Cytoplasm</td></tr>
<tr><td>19</td><td>>circRNA:chr5:128112813,128189009|circbase_id:---|type:cance|position:Exon,Intron|host_gene:SLC12A2</td><td>Nucleus</td><td>Cytoplasm</td><td>Cytoplasm</td><td>Cytoplasm</td></tr>
<tr><td>20</td><td>>circRNA:chrX:46648280,46648797|circbase_id:hsa_circ_0140330|type:normal,cancer|position:Exon,Intron|host_gene:SLC9A7</td><td>Cytoplasm</td><td>Nucleus</td><td>Cytoplasm</td><td>Cytoplasm</td></tr>
<tr><td>21</td><td>>circRNA:chr6:2107846,2110725|circbase_id:---|type:cancer|position:Intron|host_gene:GMDS</td><td>Nucleus</td><td>Nucleus</td><td>Cytoplasm</td><td>Cytoplasm</td></tr>
<tr><td>22</td><td>>circRNA:chr19:6719210,6720573|circbase_id:---|type:normal,cancer|position:Exon,Intron|host_gene:C3</td><td>Cytoplasm</td><td>Nucleus</td><td>Cytoplasm</td><td>Cytoplasm</td></tr>
<tr><td>23</td><td>>circRNA:chr12:23940217,23940376|circbase_id:---|type:cancer|position:Intron|host_gene:SOX5</td><td>Nucleus</td><td>Nucleus</td><td>Nucleus</td><td>Nucleus</td></tr>
<tr><td>24</td><td>>circRNA:chr5:179841906,179851424|circbase_id:hsa_circ_0128734|type:normal,cancer|position:Exon,Intron|host_gene:MRNIP</td><td>Nucleus</td><td>Cytoplasm</td><td>Cytoplasm</td><td>Cytoplasm</td></tr>
<tr><td>25</td><td>>circRNA:chr2:218466009,218482234|circbase_id:---|type:normal,cancer|position:Exon,Intron|host_gene:USP37</td><td>Cytoplasm</td><td>Cytoplasm</td><td>Cytoplasm</td><td>Cytoplasm</td></tr>
<tr><td>26</td><td>>circRNA:chr12:56994694,56994866|circbase_id:---|type:cancer|position:Intron|host_gene:GPR182</td><td>Nucleus</td><td>Nucleus</td><td>Nucleus</td><td>Nucleus</td></tr>
<tr><td>27</td><td>>circRNA:chr20:62162751,62172801|circbase_id:---|type:normal,cancer|position:Exon,Intron|host_gene:SS18L1</td><td>Cytoplasm</td><td>Cytoplasm</td><td>Cytoplasm</td><td>Cytoplasm</td></tr>
<tr><td>28</td><td>>circRNA:chr17:76399448,76401154|circbase_id:---|type:cancer|position:Exon,Intron|host_gene:UBE2O</td><td>Cytoplasm</td><td>Cytoplasm</td><td>Cytoplasm</td><td>Cytoplasm</td></tr>
<tr><td>29</td><td>>circRNA:chr12:57762768,57763017|circbase_id:---|type:cancer|position:Exon|host_gene:CYP27B1</td><td>Nucleus</td><td>Nucleus</td><td>Nucleus</td><td>Nucleus</td></tr>
<tr><td>30</td><td>>circRNA:chr4:20700617,20700878|circbase_id:---|type:cancer|position:Exon,Intron|host_gene:PACRGL</td><td>Nucleus</td><td>Nucleus</td><td>Nucleus</td><td>Nucleus</td></tr>
<tr><td>31</td><td>>circRNA:chr6:5255884,5256057|circbase_id:---|type:cancer|position:Intron|host_gene:LYRM4</td><td>Nucleus</td><td>Nucleus</td><td>Nucleus</td><td>Nucleus</td></tr>
<tr><td>32</td><td>>circRNA:chr3:43299753,43343478|circbase_id:---|type:normal,cancer|position:Exon,Intron|host_gene:SNRK</td><td>Cytoplasm</td><td>Cytoplasm</td><td>Cytoplasm</td><td>Cytoplasm</td></tr>
<tr><td>33</td><td>>circRNA:chr1:219179146,219193251|circbase_id:---|type:cancer|position:Exon,Intron|host_gene:LYPLAL1</td><td>Cytoplasm</td><td>Cytoplasm</td><td>Cytoplasm</td><td>Cytoplasm</td></tr>
<tr><td>34</td><td>>circRNA:chr5:131392804,131392988|circbase_id:---|type:cancer|position:Exon|host_gene:CDC42SE2</td><td>Cytoplasm</td><td>Nucleus</td><td>Nucleus</td><td>Nucleus</td></tr>
<tr><td>35</td><td>>circRNA:chr1:156946637,156947917|circbase_id:---|type:cancer|position:Exon,Intron|host_gene:ARHGEF11</td><td>Nucleus</td><td>Nucleus</td><td>Cytoplasm</td><td>Nucleus</td></tr>
<tr><td>36</td><td>>circRNA:chr1:230857474,230857807|circbase_id:---|type:cancer|position:Intron|host_gene:C1orf198</td><td>Nucleus</td><td>Nucleus</td><td>Nucleus</td><td>Nucleus</td></tr>
<tr><td>37</td><td>>circRNA:chr3:194352612,194352767|circbase_id:---|type:cancer|position:Intergenic|host_gene:---</td><td>Nucleus</td><td>Nucleus</td><td>Nucleus</td><td>Nucleus</td></tr>
<tr><td>38</td><td>>circRNA:chr2:215387004,215388211|circbase_id:---|type:normal,cancer|position:Intron|host_gene:FN1</td><td>Nucleus</td><td>Nucleus</td><td>Cytoplasm</td><td>Nucleus</td></tr>
<tr><td>39</td><td>>circRNA:chr17:16417561,16422889|circbase_id:---|type:cancer|position:Exon,Intron|host_gene:TRPV2</td><td>Cytoplasm</td><td>Cytoplasm</td><td>Cytoplasm</td><td>Cytoplasm</td></tr>
<tr><td>40</td><td>>circRNA:chr1:114756195,114756379|circbase_id:---|type:normal,cancer|position:Intron|host_gene:CSDE1</td><td>Nucleus</td><td>Nucleus</td><td>Nucleus</td><td>Nucleus</td></tr>
<tr><td>41</td><td>>circRNA:chr3:141913663,141925676|circbase_id:---|type:cancer|position:Exon,Intron|host_gene:ATP1B3</td><td>Cytoplasm</td><td>Cytoplasm</td><td>Cytoplasm</td><td>Nucleus</td></tr>
<tr><td>42</td><td>>circRNA:chr19:6690762,6692923|circbase_id:---|type:cancer|position:Intron|host_gene:C3</td><td>Nucleus</td><td>Nucleus</td><td>Cytoplasm</td><td>Nucleus</td></tr>
<tr><td>43</td><td>>circRNA:chr14:75622078,75641898|circbase_id:hsa_circ_0032691|type:normal,cancer|position:Exon,Intron|host_gene:FLVCR2</td><td>Cytoplasm</td><td>Cytoplasm</td><td>Cytoplasm</td><td>Cytoplasm</td></tr>
<tr><td>44</td><td>>circRNA:chr3:17575202,17576549|circbase_id:hsa_circ_0122770|type:cancer|position:Exon,Intron|host_gene:TBC1D5</td><td>Cytoplasm</td><td>Nucleus</td><td>Nucleus</td><td>Cytoplasm</td></tr>
<tr><td>45</td><td>>circRNA:chr6:47524167,47526740|circbase_id:---</td><td>Nucleus</td><td>Nucleus</td><td>Cytoplasm</td><td>Cytoplasm</td></tr>
</table>

	|type:cancer|position:Intron|host_gene:CD2AP				
46	>circRNA:chr13:40749137,40759106|circbase_id:---|type:normal,cancer|position:Exon,Intron|host_gene:MRPS31	Cytoplasm	Cytoplasm	Cytoplasm	Nucleus
47	>circRNA:chr18:32275546,32275773|circbase_id:---|type:cancer|position:Intron|host_gene:GAREM1	Cytoplasm	Nucleus	Nucleus	Nucleus
48	>circRNA:chr13:75814967,75815121|circbase_id:---|type:cancer|position:Intron|host_gene:LMO7	Nucleus	Nucleus	Nucleus	Nucleus
49	>circRNA:chr3:12590797,12618747|circbase_id:---|type:normal,cancer|position:Exon,Intron|host_gene:RAF1	Cytoplasm	Cytoplasm	Cytoplasm	Cytoplasm
50	>circRNA:chr9:127353845,127355472|circbase_id:---|type:cancer|position:Exon,Intron|host_gene:GARNL3	Cytoplasm	Cytoplasm	Cytoplasm	Cytoplasm
51	>circRNA:chr17:7446832,7447650|circbase_id:---|type:cancer|position:Exon,Intron|host_gene:CHRNB1	Cytoplasm	Cytoplasm	Cytoplasm	Cytoplasm
52	>circRNA:chr12:112137248,112137758|circbase_id:---|type:normal,cancer|position:Intron|host_gene:TRAFD1	Cytoplasm	Nucleus	Nucleus	Cytoplasm
53	>circRNA:chr12:124385744,124437996|circbase_id:hsa_circ_0097826|type:normal,cancer|position:Exon,Intron|host_gene:NCOR2	Cytoplasm	Cytoplasm	Cytoplasm	Cytoplasm
54	>circRNA:chr7:30023237,30023407|circbase_id:---|type:cancer|position:Intron|host_gene:FKBP14-AS1	Nucleus	Nucleus	Nucleus	Nucleus
55	>circRNA:chr20:34634614,34657244|circbase_id:---|type:normal,cancer|position:Exon,Intron|host_gene:TP53INP2	Cytoplasm	Cytoplasm	Cytoplasm	Cytoplasm
56	>circRNA:chr2:161318821,161395203|circbase_id:---|type:normal,cancer|position:Exon,Intron|host_gene:PSMD14	Cytoplasm	Cytoplasm	Cytoplasm	Cytoplasm
57	>circRNA:chr19:41302949,41303945|circbase_id:---|type:normal,cancer|position:Intron|host_gene:HNRNPUL1	Nucleus	Nucleus	Cytoplasm	Cytoplasm
58	>circRNA:chr7:143294229,143303933|circbase_id:---|type:normal,cancer|position:Exon,Intron|host_gene:CASP2	Cytoplasm	Cytoplasm	Cytoplasm	Cytoplasm
59	>circRNA:chr4:159279045,159279144|circbase_id:---|type:cancer|position:Intron|host_gene:RAPGEF2	Nucleus	Nucleus	Nucleus	Nucleus
60	>circRNA:chr6:149572506,149582603|circbase_id:hsa_circ_0078212|type:normal,cancer|position:Exon,Intron|host_gene:GINM1	Nucleus	Cytoplasm	Cytoplasm	Cytoplasm
61	>circRNA:chr4:186602903,186636914|circbase_id:---|type:normal,cancer|position:Exon,Intron|host_gene:FAT1	Cytoplasm	Cytoplasm	Cytoplasm	Cytoplasm
62	>circRNA:chr4:82862639,82863317|circbase_id:---|type:normal,cancer|position:Exon,Intron|host_gene:SEC31A	Nucleus	Nucleus	Nucleus	Nucleus
63	>circRNA:chr2:164699404,164730115|circbase_id:---|type:normal,cancer|position:Exon,Intron|host_gene:COBLL1	Cytoplasm	Cytoplasm	Cytoplasm	Cytoplasm
64	>circRNA:chr2:230474393,230494460|circbase_id:---|type:cancer|position:Exon,Intron|host_gene:SP100	Cytoplasm	Cytoplasm	Nucleus	Cytoplasm
65	>circRNA:chr2:43875145,43878514|circbase_id:---|type:normal,cancer|position:Exon,Intron|host_gene:ABCG8	Cytoplasm	Cytoplasm	Cytoplasm	Cytoplasm
66	>circRNA:chr2:241343093,241343724|circbase_id:---|type:cancer|position:Intron|host_gene:SEPTIN2	Nucleus	Nucleus	Nucleus	Nucleus
67	>circRNA:chr14:55276110,55276222|circbase_id:---|type:normal,cancer|position:Intron|host_gene:FBXO34	Cytoplasm	Nucleus	Nucleus	Nucleus
68	>circRNA:chr22:37812854,37813609|circbase_id:---|type:normal,cancer|position:Exon,Intron|host_gene:GCAT	Nucleus	Nucleus	Cytoplasm	Cytoplasm
69	>circRNA:chr1:161223349,161223425|circbase_id:---|type:cancer|position:Exon|host_gene:APOA2	Cytoplasm	Nucleus	Nucleus	Nucleus
70	>circRNA:chr16:677754,678012|circbase_id:---|type:cancer|position:Exon|host_gene:RHBDL1	Nucleus	Nucleus	Nucleus	Nucleus
71	>circRNA:chr13:102793340,102797009|circbase_id:hsa_circ_0099796|type:normal,cancer|position:Exon,Intron|host_gene:POGLUT2	Nucleus	Cytoplasm	Cytoplasm	Cytoplasm
72	>circRNA:chr1:10312702,10313297|circbase_id:---|type:cancer|position:Intron|host_gene:KIF1B	Nucleus	Nucleus	Nucleus	Nucleus
73	>circRNA:chr14:45148836,45189362|circbase_id:---|type:normal,cancer|position:Exon,Intron|host_gene:FANCM	Nucleus	Cytoplasm	Cytoplasm	Cytoplasm
74	>circRNA:chr13:23365165,23371165|circbase_id:hsa_circ_0002046|type:normal,cancer|position:Exon,Intron|host_gene:SACS	Cytoplasm	Cytoplasm	Cytoplasm	Cytoplasm
75	>circRNA:chr7:107211098,107211265|circbase_id:---|type:cancer|position:Exon,Intron|host_gene:COG5	Cytoplasm	Nucleus	Nucleus	Nucleus
76	>circRNA:chr19:45553960,45554513|circbase_id:---|type:cancer|position:Intron|host_gene:OPA3	Nucleus	Nucleus	Nucleus	Cytoplasm
77	>circRNA:chr16:391766,392121|circbase_id:---|type:normal,cancer|position:Exon,Intron|host_gene:RPL23AP5	Nucleus	Nucleus	Nucleus	Cytoplasm
78	>circRNA:chr2:63000099,63000222|circbase_id:---|type:normal,cancer|position:Intron|host_gene:EHBP1	Nucleus	Nucleus	Nucleus	Nucleus
79	>circRNA:chr1:63531819,63534216|circbase_id:hsa_circ_0113871|type:normal,cancer|position:Exon,Intron|host_gene:EFCAB7	Nucleus	Cytoplasm	Cytoplasm	Cytoplasm
80	>circRNA:chr5:127534750,127535060|circbase_id:---|type:cancer|position:Intron|host_gene:PRRC1	Nucleus	Nucleus	Nucleus	Nucleus
81	>circRNA:chr12:6550940,6551503|circbase_id:---|type:normal,cancer|position:Exon,Intron|host_gene:IFFO1	Nucleus	Nucleus	Nucleus	Cytoplasm
82	>circRNA:chr6:138480311,138483095|circbase_id:---|type:normal,cancer|position:Intron|host_gene:NHSL1	Nucleus	Cytoplasm	Cytoplasm	Cytoplasm
83	>circRNA:chr1:215586494,215619291|circbase_id:---|type:normal,cancer|position:Exon,Intron|host_gene:KCTD3	Cytoplasm	Cytoplasm	Cytoplasm	Cytoplasm
84	>circRNA:chr16:70466881,70469323|circbase_id:hsa_circ_0105862|type:normal,cancer|position:Exon,Intron|host_gene:FCSK	Nucleus	Nucleus	Cytoplasm	Nucleus
85	>circRNA:chr14:77552095,77597380|circbase_id:hsa_circ_0032787|type:normal,cancer|position:Exon,Intron|host_gene:SPTLC2	Cytoplasm	Cytoplasm	Cytoplasm	Cytoplasm
86	>circRNA:chr20:58429752,58434705|circbase_id:---|type:normal,cancer|position:Exon,Intron|host_gene:VAPB	Nucleus	Cytoplasm	Cytoplasm	Cytoplasm
87	>circRNA:chr2:73224971,73225185|circbase_id:---|type:cancer|position:Intron|host_gene:SMYD5	Nucleus	Nucleus	Nucleus	Nucleus
88	>circRNA:chr4:87132270,87134694|circbase_id:---|type:cancer|position:Exon,Intron|host_gene:AFF1	Cytoplasm	Nucleus	Cytoplasm	Cytoplasm
89	>circRNA:chr10:124039357,124039427|circbase_id:---|type:cancer|position:Intron|host_gene:CHST15	Nucleus	Nucleus	Cytoplasm	Cytoplasm
90	>circRNA:chr14:22973149,22978421|circbase_id:---|type:cancer|position:Intron|host_gene:AL132780.3	Cytoplasm	Cytoplasm	Cytoplasm	Cytoplasm
91	>circRNA:chr6:43090512,43090869|circbase_id:---|type:cancer|position:Intron|host_gene:PTK7	Nucleus	Nucleus	Nucleus	Nucleus
92	>circRNA:chr21:33575697,33579562|circbase_id:---|type:normal,cancer|position:Exon,Intron|host_gene:DONSON	Cytoplasm	Cytoplasm	Cytoplasm	Cytoplasm
93	>circRNA:chr5:34006528,34006628|circbase_id:---	Nucleus	Nucleus	Nucleus	Nucleus

	|type:normal,cancer|position:Intron|host_gene:AMACR				
94	>circRNA:chr19:19647128,19647432|circbase_id:---|type:cancer|position:Exon|host_gene:ATP13A1	Nucleus	Nucleus	Cytoplasm	Nucleus
95	>circRNA:chr12:79940361,79940655|circbase_id:---|type:cancer|position:Exon,Intron|host_gene:PPP1R12A-AS1	Cytoplasm	Nucleus	Cytoplasm	Cytoplasm
96	>circRNA:chr1:99870757,99884451|circbase_id:---|type:cancer|position:Exon,Intron|host_gene:AGL	Nucleus	Cytoplasm	Cytoplasm	Cytoplasm
97	>circRNA:chr1:28031282,28032016|circbase_id:---|type:cancer|position:Intron|host_gene:EYA3	Nucleus	Nucleus	Nucleus	Nucleus
98	>circRNA:chr11:679687,686997|circbase_id:---|type:normal,cancer|position:Exon,Intron|host_gene:DEAF1	Cytoplasm	Cytoplasm	Cytoplasm	Cytoplasm
99	>circRNA:chr22:43932877,43937272|circbase_id:hsa_circ_0063698|type:normal,cancer|position:Exon,Intron|host_gene:PNPLA3	Cytoplasm	Cytoplasm	Cytoplasm	Cytoplasm
100	>circRNA:chr7:101233135,101233212|circbase_id:---|type:cancer|position:Intron|host_gene:CLDN15	Nucleus	Nucleus	Nucleus	Nucleus
101	>circRNA:chrX:19354544,19355726|circbase_id:---|type:normal,cancer|position:Exon,Intron|host_gene:PDHA1	Cytoplasm	Cytoplasm	Cytoplasm	Cytoplasm
102	>circRNA:chr12:19462509,19518113|circbase_id:---|type:normal,cancer|position:Exon,Intron|host_gene:AEBP2	Cytoplasm	Cytoplasm	Cytoplasm	Cytoplasm
103	>circRNA:chr22:38071744,38072457|circbase_id:---|type:cancer|position:Intron|host_gene:PICK1	Nucleus	Nucleus	Cytoplasm	Cytoplasm
104	>circRNA:chr18:35977435,35977600|circbase_id:---|type:cancer|position:Exon|host_gene:C18orf21	Cytoplasm	Nucleus	Cytoplasm	Nucleus
105	>circRNA:chr21:36333645,36372531|circbase_id:hsa_circ_0061614|type:normal,cancer|position:Exon,Intron|host_gene:MORC3	Cytoplasm	Cytoplasm	Cytoplasm	Nucleus
106	>circRNA:chr15:40602914,40615378|circbase_id:---|type:normal,cancer|position:Exon,Intron|host_gene:KNL1	Cytoplasm	Cytoplasm	Cytoplasm	Nucleus
107	>circRNA:chr2:155838400,155840984|circbase_id:---|type:cancer|position:Intergenic|host_gene:---	Nucleus	Nucleus	Cytoplasm	Cytoplasm
108	>circRNA:chr5:79300152,79312498|circbase_id:---|type:normal,cancer|position:Exon,Intron|host_gene:JMY	Cytoplasm	Cytoplasm	Cytoplasm	Cytoplasm
109	>circRNA:chr2:159398828,159400664|circbase_id:---|type:normal,cancer|position:Exon,Intron|host_gene:BAZ2B	Cytoplasm	Cytoplasm	Cytoplasm	Nucleus
110	>circRNA:chr19:54453661,54454267|circbase_id:---|type:normal,cancer|position:Intron|host_gene:LENG8	Nucleus	Nucleus	Nucleus	Cytoplasm
111	>circRNA:chr11:59639047,59641585|circbase_id:---|type:normal,cancer|position:Exon,Intron|host_gene:PATL1	Nucleus	Cytoplasm	Cytoplasm	Cytoplasm
112	>circRNA:chr19:17935191,17936385|circbase_id:---|type:normal,cancer|position:Exon,Intron|host_gene:CCDC124	Nucleus	Cytoplasm	Cytoplasm	Cytoplasm
113	>circRNA:chr1:31004012,31007102|circbase_id:---|type:normal,cancer|position:Exon,Intron|host_gene:PUM1	Nucleus	Nucleus	Cytoplasm	Cytoplasm
114	>circRNA:chr1:9595889,9602336|circbase_id:---|type:cancer|position:Exon,Intron|host_gene:TMEM201	Nucleus	Cytoplasm	Cytoplasm	Cytoplasm
115	>circRNA:chr10:96142830,96151566|circbase_id:---|type:normal,cancer|position:Exon,Intron|host_gene:ZNF518A	Nucleus	Cytoplasm	Cytoplasm	Cytoplasm
116	>circRNA:chr16:30942115,30942536|circbase_id:hsa_circ_0039122|type:normal,cancer |position:Exon,Intron|host_gene:FBXL19	Cytoplasm	Cytoplasm	Cytoplasm	Cytoplasm
117	>circRNA:chr15:64848592,64848742|circbase_id:---|type:normal,cancer|position:Exon,Intron|host_gene:PLEKHO2	Cytoplasm	Nucleus	Nucleus	Cytoplasm
118	>circRNA:chr15:34243973,34245867|circbase_id:hsa_circ_0103320|type:normal,cancer |position:Exon,Intron|host_gene:SLC12A6	Nucleus	Nucleus	Cytoplasm	Cytoplasm
119	>circRNA:chr5:1334288,1339005|circbase_id:---|type:normal,cancer|position:Exon,Intron|host_gene:CLPTM1L	Cytoplasm	Cytoplasm	Cytoplasm	Cytoplasm
120	>circRNA:chr3:9464424,9465558|circbase_id:---|type:normal,cancer|position:Exon,Intron|host_gene:SETD5	Nucleus	Nucleus	Cytoplasm	Cytoplasm
121	>circRNA:chr15:85724378,85724700|circbase_id:---|type:cancer|position:Intron|host_gene:AKAP13	Nucleus	Nucleus	Nucleus	Nucleus
122	>circRNA:chr7:45892060,45892937|circbase_id:---|type:cancer|position:Intron|host_gene:IGFBP1	Nucleus	Nucleus	Cytoplasm	Nucleus
123	>circRNA:chr1:201847278,201853097|circbase_id:--- |type:normal,cancer|position:Exon,Intron|host_gene:IPO9	Cytoplasm	Cytoplasm	Cytoplasm	Cytoplasm
124	>circRNA:chr8:98108435,98108950|circbase_id:---|type:cancer|position:Exon,Intron|host_gene:RIDA	Nucleus	Nucleus	Nucleus	Nucleus
125	>circRNA:chr4:73412131,73413356|circbase_id:---|type:cancer|position:Exon,Intron|host_gene:ALB	Nucleus	Nucleus	Cytoplasm	Cytoplasm
126	>circRNA:chr3:62275872,62282869|circbase_id:---|type:normal,cancer|position:Exon,Intron|host_gene:PTPRG	Cytoplasm	Cytoplasm	Cytoplasm	Cytoplasm
127	>circRNA:chr6:31533567,31533795|circbase_id:---|type:cancer|position:Exon|host_gene:DDX39B	Nucleus	Nucleus	Nucleus	Nucleus
128	>circRNA:chr12:96925601,96925753|circbase_id:---|type:cancer|position:Intron|host_gene:NEDD1	Nucleus	Nucleus	Nucleus	Nucleus
129	>circRNA:chr2:26040120,26040763|circbase_id:---|type:cancer|position:Intron|host_gene:RAB10	Nucleus	Nucleus	Nucleus	Nucleus
130	>circRNA:chr10:103139405,103181328|circbase_id:hsa_circ_0092515|type:normal,cancer|position:Exon,Intron|host_gene:NT5C2	Cytoplasm	Cytoplasm	Cytoplasm	Cytoplasm
131	>circRNA:chr4:123118758,123122283|circbase_id:---|type:cancer|position:Intron|host_gene:SPATA5	Nucleus	Nucleus	Cytoplasm	Nucleus
132	>circRNA:chr9:111363587,111390101|circbase_id:---|type:normal,cancer|position:Exon,Intron|host_gene:ECPAS	Cytoplasm	Cytoplasm	Cytoplasm	Cytoplasm
133	>circRNA:chr12:117064326,117064473|circbase_id:---|type:cancer|position:Intron|host_gene:TESC	Nucleus	Nucleus	Nucleus	Nucleus
134	>circRNA:chr7:131443480,131449243|circbase_id:---|type:cancer|position:Exon,Intron|host_gene:MKLN1	Nucleus	Nucleus	Cytoplasm	Cytoplasm
135	>circRNA:chr10:3780105,3782214|circbase_id:---|type:normal,cancer|position:Exon,Intron|host_gene:KLF6	Cytoplasm	Cytoplasm	Cytoplasm	Cytoplasm
136	>circRNA:chr14:20693650,20693757|circbase_id:---|type:cancer|position:Intron|host_gene:EGILA	Nucleus	Nucleus	Nucleus	Cytoplasm
137	>circRNA:chr8:144430535,144430652|circbase_id:---|type:cancer|position:Exon,Intron|host_gene:TONSL	Nucleus	Nucleus	Nucleus	Nucleus
138	>circRNA:chr9:71684685,71684823|circbase_id:---|type:cancer|position:Exon|host_gene:CEMIP2	Nucleus	Nucleus	Nucleus	Cytoplasm
139	>circRNA:chr1:110370791,110370989|circbase_id:---|type:cancer|position:Intron|host_gene:LAMTOR5-AS1	Nucleus	Nucleus	Nucleus	Nucleus
140	>circRNA:chr1:156731749,156732129|circbase_id:---|type:cancer|position:Exon,Intron|host_gene:METTL25B	Nucleus	Nucleus	Nucleus	Nucleus
141	>circRNA:chr12:121318276,121320459|circbase_id:---	Nucleus	Cytoplasm	Cytoplasm	Cytoplasm

<table>
<tr><td></td><td>|type:normal,cancer|position:Exon,Intron|host_gene:ANAPC5</td><td></td><td></td><td></td><td></td></tr>
<tr><td>142</td><td>>circRNA:chr6:121205074,121321794|circbase_id:---
|type:normal,cancer|position:Exon,Intron|host_gene:TBC1D32</td><td>Nucleus</td><td>Cytoplasm</td><td>Cytoplasm</td><td>Cytoplasm</td></tr>
<tr><td>143</td><td>>circRNA:chr6:4779493,4780044|circbase_id:---
|type:cancer|position:Intron|host_gene:CDYL</td><td>Nucleus</td><td>Nucleus</td><td>Nucleus</td><td>Nucleus</td></tr>
<tr><td>144</td><td>>circRNA:chr2:218812221,218815147|circbase_id:--- |type:normal,cancer
|position:Exon,Intron|host_gene:CYP27A1</td><td>Nucleus</td><td>Cytoplasm</td><td>Cytoplasm</td><td>Cytoplasm</td></tr>
<tr><td>145</td><td>>circRNA:chr20:5783038,5783138|circbase_id:---
|type:cancer|position:Intron|host_gene:SHLD1</td><td>Nucleus</td><td>Nucleus</td><td>Nucleus</td><td>Nucleus</td></tr>
<tr><td>146</td><td>>circRNA:chr18:34893159,34902932|circbase_id:---
|type:cancer|position:Exon,Intron|host_gene:AC068506.1</td><td>Nucleus</td><td>Cytoplasm</td><td>Cytoplasm</td><td>Cytoplasm</td></tr>
<tr><td>147</td><td>>circRNA:chr9:93247542,93253082|circbase_id:---
|type:normal,cancer|position:Exon,Intron|host_gene:WNK2</td><td>Cytoplasm</td><td>Cytoplasm</td><td>Cytoplasm</td><td>Cytoplasm</td></tr>
<tr><td>148</td><td>>circRNA:chr15:52042753,52050137|circbase_id:---
|type:normal,cancer|position:Exon,Intron|host_gene:MAPK6</td><td>Nucleus</td><td>Cytoplasm</td><td>Cytoplasm</td><td>Cytoplasm</td></tr>
<tr><td>149</td><td>>circRNA:chr4:4239914,4246343|circbase_id:---
|type:normal,cancer|position:Exon,Intron|host_gene:TMEM128</td><td>Nucleus</td><td>Cytoplasm</td><td>Cytoplasm</td><td>Cytoplasm</td></tr>
<tr><td>150</td><td>>circRNA:chr17:38720747,38721806|circbase_id:---|type:normal,cancer
|position:Intron|host_gene:MLLT6</td><td>Nucleus</td><td>Nucleus</td><td>Cytoplasm</td><td>Nucleus</td></tr>
<tr><td>151</td><td>>circRNA:chr2:95165058,95165403|circbase_id:---
|type:cancer|position:Exon|host_gene:ZNF514</td><td>Cytoplasm</td><td>Nucleus</td><td>Cytoplasm</td><td>Nucleus</td></tr>
<tr><td>152</td><td>>circRNA:chr17:78826111,78826819|circbase_id:---
|type:cancer|position:Exon,Intron|host_gene:USP36</td><td>Nucleus</td><td>Nucleus</td><td>Nucleus</td><td>Cytoplasm</td></tr>
<tr><td>153</td><td>>circRNA:chr2:238530162,238533716|circbase_id:---
|type:cancer|position:Intron|host_gene:LINC01107</td><td>Nucleus</td><td>Cytoplasm</td><td>Cytoplasm</td><td>Nucleus</td></tr>
<tr><td>154</td><td>>circRNA:chr8:29051343,29051482|circbase_id:---
|type:cancer|position:Exon|host_gene:HMBOX1</td><td>Nucleus</td><td>Nucleus</td><td>Nucleus</td><td>Nucleus</td></tr>
<tr><td>155</td><td>>circRNA:chr8:30396474,30396595|circbase_id:---
|type:cancer|position:Intron|host_gene:RBPMS</td><td>Nucleus</td><td>Nucleus</td><td>Nucleus</td><td>Nucleus</td></tr>
<tr><td>156</td><td>>circRNA:chr17:4191186,4192860|circbase_id:hsa_circ_0106898|type:no
rmal,cancer |position:Exon,Intron|host_gene:ANKFY1</td><td>Nucleus</td><td>Nucleus</td><td>Cytoplasm</td><td>Cytoplasm</td></tr>
<tr><td>157</td><td>>circRNA:chr15:35209925,35214347|circbase_id:---
|type:cancer|position:Intron|host_gene:ANP32AP1</td><td>Nucleus</td><td>Cytoplasm</td><td>Cytoplasm</td><td>Cytoplasm</td></tr>
<tr><td>158</td><td>>circRNA:chr7:111895611,111945798|circbase_id:hsa_circ_0133059|type
:normal,cancer|position:Exon,Intron|host_gene:DOCK4</td><td>Cytoplasm</td><td>Cytoplasm</td><td>Cytoplasm</td><td>Cytoplasm</td></tr>
<tr><td>159</td><td>>circRNA:chr5:138556784,138557107|circbase_id:---
|type:cancer|position:Exon|host_gene:HSPA9</td><td>Nucleus</td><td>Nucleus</td><td>Nucleus</td><td>Nucleus</td></tr>
<tr><td>160</td><td>>circRNA:chr17:15537557,15538296|circbase_id:---
|type:cancer|position:Intron|host_gene:TVP23C-CDRT4</td><td>Nucleus</td><td>Nucleus</td><td>Nucleus</td><td>Cytoplasm</td></tr>
<tr><td>161</td><td>>circRNA:chr16:87308532,87309254|circbase_id:---
|type:normal,cancer|position:Intron|host_gene:C16orf95</td><td>Cytoplasm</td><td>Cytoplasm</td><td>Cytoplasm</td><td>Cytoplasm</td></tr>
<tr><td>162</td><td>>circRNA:chr5:88180223,88180513|circbase_id:---
|type:cancer|position:Intergenic|host_gene:---</td><td>Nucleus</td><td>Nucleus</td><td>Nucleus</td><td>Nucleus</td></tr>
<tr><td>163</td><td>>circRNA:chr2:147952372,147975975|circbase_id:---
|type:normal,cancer|position:Exon,Intron|host_gene:ORC4</td><td>Cytoplasm</td><td>Cytoplasm</td><td>Cytoplasm</td><td>Cytoplasm</td></tr>
<tr><td>164</td><td>>circRNA:chr1:119645519,119645641|circbase_id:---
|type:cancer|position:Intron|host_gene:ZNF697</td><td>Cytoplasm</td><td>Nucleus</td><td>Nucleus</td><td>Nucleus</td></tr>
<tr><td>165</td><td>>circRNA:chr13:75288933,75312898|circbase_id:---
|type:normal,cancer|position:Exon,Intron|host_gene:TBC1D4</td><td>Cytoplasm</td><td>Cytoplasm</td><td>Cytoplasm</td><td>Cytoplasm</td></tr>
<tr><td>166</td><td>>circRNA:chr9:35100931,35101962|circbase_id:---
|type:cancer|position:Exon,Intron|host_gene:STOML2</td><td>Cytoplasm</td><td>Cytoplasm</td><td>Cytoplasm</td><td>Cytoplasm</td></tr>
<tr><td>167</td><td>>circRNA:chr11:47821723,47840065|circbase_id:---
|type:normal,cancer|position:Exon,Intron|host_gene:NUP160</td><td>Cytoplasm</td><td>Cytoplasm</td><td>Cytoplasm</td><td>Cytoplasm</td></tr>
<tr><td>168</td><td>>circRNA:chr16:66729039,66732505|circbase_id:---
|type:normal,cancer|position:Exon,Intron|host_gene:DYNC1LI2</td><td>Nucleus</td><td>Cytoplasm</td><td>Cytoplasm</td><td>Cytoplasm</td></tr>
<tr><td>169</td><td>>circRNA:chr1:61255735,61264793|circbase_id:---
|type:cancer|position:Intron|host_gene:NFIA</td><td>Nucleus</td><td>Nucleus</td><td>Cytoplasm</td><td>Nucleus</td></tr>
<tr><td>170</td><td>>circRNA:chr1:61220846,61221205|circbase_id:---
|type:cancer|position:Intron|host_gene:NFIA</td><td>Nucleus</td><td>Nucleus</td><td>Nucleus</td><td>Nucleus</td></tr>
<tr><td>171</td><td>>circRNA:chr17:49044527,49044908|circbase_id:---
|type:cancer|position:Intron|host_gene:IGF2BP1</td><td>Nucleus</td><td>Nucleus</td><td>Nucleus</td><td>Nucleus</td></tr>
<tr><td>172</td><td>>circRNA:chr8:1786337,1786461|circbase_id:---
|type:cancer|position:Exon|host_gene:CLN8</td><td>Nucleus</td><td>Nucleus</td><td>Nucleus</td><td>Nucleus</td></tr>
<tr><td>173</td><td>>circRNA:chr4:99608776,99613030|circbase_id:---
|type:cancer|position:Exon,Intron|host_gene:MTTP</td><td>Cytoplasm</td><td>Cytoplasm</td><td>Cytoplasm</td><td>Cytoplasm</td></tr>
<tr><td>174</td><td>>circRNA:chr9:100264821,100270046|circbase_id:---
|type:normal,cancer|position:Exon,Intron|host_gene:INVS</td><td>Nucleus</td><td>Cytoplasm</td><td>Cytoplasm</td><td>Nucleus</td></tr>
<tr><td>175</td><td>>circRNA:chr10:50460320,50460377|circbase_id: ---
|type:cancer|position:Intron|host_gene:SGMS1</td><td>Cytoplasm</td><td>Nucleus</td><td>Nucleus</td><td>Nucleus</td></tr>
<tr><td>176</td><td>>circRNA:chr3:123126175,123126550|circbase_id:---
|type:cancer|position:Intron|host_gene:PDIA5</td><td>Nucleus</td><td>Nucleus</td><td>Nucleus</td><td>Nucleus</td></tr>
<tr><td>177</td><td>>circRNA:chr17:59685586,59693839|circbase_id:---
|type:cancer|position:Exon,Intron|host_gene:CLTC</td><td>Nucleus</td><td>Cytoplasm</td><td>Cytoplasm</td><td>Cytoplasm</td></tr>
<tr><td>178</td><td>>circRNA:chr10:114970440,114972046|circbase_id:---
|type:cancer|position:Intron|host_gene:TRUB1</td><td>Nucleus</td><td>Nucleus</td><td>Cytoplasm</td><td>Nucleus</td></tr>
<tr><td>179</td><td>>circRNA:chr4:109690849,109694700|circbase_id:hsa_circ_0070663|typ
e:normal,cancer|position:Exon,Intron|host_gene:CASP6</td><td>Cytoplasm</td><td>Cytoplasm</td><td>Cytoplasm</td><td>Cytoplasm</td></tr>
<tr><td>180</td><td>>circRNA:chr11:61419921,61421608|circbase_id:---
|type:cancer|position:Exon,Intron|host_gene:CPSF7</td><td>Nucleus</td><td>Cytoplasm</td><td>Cytoplasm</td><td>Cytoplasm</td></tr>
<tr><td>181</td><td>>circRNA:chr3:71068164,71068276|circbase_id:---
cancer|type:|position:Intron|host_gene:FOXP1</td><td>Nucleus</td><td>Nucleus</td><td>Nucleus</td><td>Nucleus</td></tr>
<tr><td>182</td><td>>circRNA:chr21:41338377,41348724|circbase_id:---
|type:cancer|position:Exon,Intron|host_gene:FAM3B</td><td>Cytoplasm</td><td>Cytoplasm</td><td>Cytoplasm</td><td>Cytoplasm</td></tr>
<tr><td>183</td><td>>circRNA:chr14:103405075,103413800|circbase_id:---
|type:normal,cancer|position:Exon,Intron|host_gene:MARK3</td><td>Nucleus</td><td>Cytoplasm</td><td>Cytoplasm</td><td>Cytoplasm</td></tr>
<tr><td>184</td><td>>circRNA:chr6:131722993,131723127|circbase_id:---
|type:cancer|position:Intron|host_gene:ENPP3</td><td>Cytoplasm</td><td>Nucleus</td><td>Nucleus</td><td>Nucleus</td></tr>
<tr><td>185</td><td>>circRNA:chr4:183663846,183668117|circbase_id:---
|type:normal,cancer|position:Exon,Intron|host_gene:TRAPPC11</td><td>Cytoplasm</td><td>Cytoplasm</td><td>Cytoplasm</td><td>Cytoplasm</td></tr>
<tr><td>186</td><td>>circRNA:chr2:164789284,164789542|circbase_id:---
|type:cancer|position:Intron|host_gene:COBLL1</td><td>Nucleus</td><td>Nucleus</td><td>Nucleus</td><td>Nucleus</td></tr>
<tr><td>187</td><td>>circRNA:chr20:25455672,25477089|circbase_id:---
|type:normal,cancer|position:Exon,Intron|host_gene:NINL</td><td>Nucleus</td><td>Cytoplasm</td><td>Cytoplasm</td><td>Cytoplasm</td></tr>
<tr><td>188</td><td>>circRNA:chr3:121948172,121948679|circbase_id:---
|type:cancer|position: Intergenic|host_gene:---</td><td>Nucleus</td><td>Nucleus</td><td>Nucleus</td><td>Nucleus</td></tr>
<tr><td>189</td><td>>circRNA:chr10:20290435,20290592|circbase_id:---</td><td>Nucleus</td><td>Nucleus</td><td>Nucleus</td><td>Nucleus</td></tr>
</table>

	|type:cancer|position:Intergenic|host_gene:---				
190	>circRNA:chr19:34959295,34959449|circbase_id:--- |type:cancer|position:Exon|host_gene:ZNF792	Cytoplasm	Nucleus	Nucleus	Nucleus
191	>circRNA:chr1:147161711,147166939|circbase_id:hsa_circ_0013943|type:normal,cancer|position:Exon,Intron|host_gene:PRKAB2	Nucleus	Cytoplasm	Cytoplasm	Cytoplasm
192	>circRNA:chr20:35012597,35013066|circbase_id:--- |type:cancer|position:Exon,Intron|host_gene:TRPC4AP	Cytoplasm	Cytoplasm	Cytoplasm	Cytoplasm
193	>circRNA:chr1:91734659,91734775|circbase_id:--- |type:cancer|position:Intron|host_gene:TGFBR3	Nucleus	Nucleus	Nucleus	Nucleus
194	>circRNA:chr12:46775071,46775225|circbase_id:--- |type:cancer|position:Exon,Intron|host_gene:SLC38A4	Nucleus	Nucleus	Nucleus	Nucleus
195	>circRNA:chr9:5665677,5666072|circbase_id:--- |type:cancer|position:Intron|host_gene:RIC1	Nucleus	Nucleus	Cytoplasm	Cytoplasm
196	>circRNA:chr12:120514704,120514881|circbase_id:--- |type:cancer|position:Intron|host_gene:COQ5	Nucleus	Nucleus	Nucleus	Nucleus
197	>circRNA:chr14:89481357,89486671|circbase_id:--- |type:cancer|position:Exon,Intron|host_gene:FOXN3	Nucleus	Cytoplasm	Cytoplasm	Cytoplasm
198	>circRNA:chr16:9106860,9107658|circbase_id:--- |type:cancer|position:Exon|host_gene:C16orf72	Nucleus	Nucleus	Nucleus	Cytoplasm
199	>circRNA:chr3:63834062,63838499|circbase_id:hsa_circ_0124444|type:normal,cancer |position:Exon,Intron|host_gene:THOC7	Nucleus	Cytoplasm	Cytoplasm	Cytoplasm
200	>circRNA:chr12:12232862,12233024|circbase_id:--- |type:cancer|position:Intron|host_gene:LRP6	Nucleus	Nucleus	Nucleus	Nucleus
201	>circRNA:chr12:44187723,44188132|circbase_id:--- |type:cancer|position:Intron|host_gene:TMEM117	Nucleus	Nucleus	Nucleus	Nucleus
202	>circRNA:chr9:71887216,71887370|circbase_id:--- |type:cancer|position:Intron|host_gene:ABHD17B	Nucleus	Nucleus	Nucleus	Nucleus
203	>circRNA:chr22:24119366,24134415|circbase_id:hsa_circ_0062595|type:normal,cancer|position:Exon,Intron|host_gene:CABIN1	Cytoplasm	Cytoplasm	Cytoplasm	Cytoplasm
204	>circRNA:chr11:61282788,61282905|circbase_id:--- |type:cancer|position:Exon,Intron|host_gene:VWCE	Cytoplasm	Nucleus	Nucleus	Nucleus
205	>circRNA:chr17:38684711,38686901|circbase_id:--- |type:normal,cancer|position:Intergenic|host_gene:---	Nucleus	Nucleus	Cytoplasm	Cytoplasm
206	>circRNA:chr2:77643040,77643603|circbase_id:--- |type:cancer|position:Intergenic|host_gene:---	Nucleus	Nucleus	Nucleus	Nucleus
207	>circRNA:chr17:18028250,18028345|circbase_id:--- |type:cancer|position:Exon|host_gene:ATPAF2	Cytoplasm	Nucleus	Nucleus	Cytoplasm
208	>circRNA:chr2:241809781,241809925|circbase_id:--- |type:cancer|position:Exon,Intron|host_gene:AC131097.2	Nucleus	Nucleus	Nucleus	Cytoplasm
209	>circRNA:chr10:119847612,119859884|circbase_id:hsa_circ_0092916|type:normal,cancer|position:Exon,Intron|host_gene:MCMBP	Cytoplasm	Cytoplasm	Cytoplasm	Cytoplasm
210	>circRNA:chr12:40095464,40095584|circbase_id:--- |type:cancer|position:Intron|host_gene:SLC2A13	Nucleus	Nucleus	Nucleus	Nucleus
211	>circRNA:chr14:74943785,74944379|circbase_id:--- |type:cancer|position:Intron|host_gene:PGF	Cytoplasm	Nucleus	Nucleus	Cytoplasm
212	>circRNA:chr9:104096147,104098568|circbase_id:--- |type:cancer|position:Exon,Intron|host_gene:SMC2	Nucleus	Nucleus	Nucleus	Cytoplasm
213	>circRNA:chr1:17413536,17425684|circbase_id:--- |type:normal,cancer|position:Exon,Intron|host_gene:RCC2	Cytoplasm	Cytoplasm	Cytoplasm	Cytoplasm
214	>circRNA:chr6:52010308,52035721|circbase_id:--- |type:normal,cancer|position:Exon,Intron|host_gene:PKHD1	Nucleus	Cytoplasm	Cytoplasm	Cytoplasm
215	>circRNA:chr20:63196004,63196108|circbase_id:--- |type:cancer|position:Exon|host_gene:YTHDF1	Nucleus	Nucleus	Nucleus	Nucleus
216	>circRNA:chr6:128184431,128185625|circbase_id:--- |type:normal,cancer|position:Exon,Intron|host_gene:PTPRK	Nucleus	Cytoplasm	Cytoplasm	Cytoplasm
217	>circRNA:chr10:95610196,95643206|circbase_id:--- |type:cancer|position:Exon,Intron|host_gene:ALDH18A1	Cytoplasm	Cytoplasm	Cytoplasm	Cytoplasm
218	>circRNA:chr14:39405341,39405781|circbase_id:--- |type:cancer|position:Intron|host_gene:FBXO33	Nucleus	Nucleus	Nucleus	Nucleus
219	>circRNA:chr4:105937008,105942702|circbase_id:hsa_circ_0124953|type:normal,cancer|position:Exon,Intron|host_gene:NPNT	Cytoplasm	Cytoplasm	Cytoplasm	Cytoplasm
220	>circRNA:chr1:2106539,2106913|circbase_id:--- |type:cancer|position:Intron|host_gene:PRKCZ	Nucleus	Nucleus	Cytoplasm	Nucleus
221	>circRNA:chr4:26978611,26979020|circbase_id:--- |type:cancer|position:Intron|host_gene:STIM2	Nucleus	Nucleus	Nucleus	Nucleus
222	>circRNA:chr11:9724657,9732710|circbase_id:hsa_circ_0021209|type:normal,cancer |position:Exon,Intron|host_gene:SWAP70	Cytoplasm	Cytoplasm	Cytoplasm	Cytoplasm
223	>circRNA:chr5:109713519,109847790|circbase_id:--- |type:normal,cancer|position:Exon,Intron|host_gene:MAN2A1	Cytoplasm	Cytoplasm	Cytoplasm	Nucleus
224	>circRNA:chr7:6733398,6735389|circbase_id:--- |type:normal,cancer|position:Exon,Intron|host_gene:PMS2	Nucleus	Nucleus	Nucleus	Cytoplasm
225	>circRNA:chr1:162216284,162216435|circbase_id:--- |type:cancer|position:Intron|host_gene:NOS1AP	Nucleus	Nucleus	Nucleus	Nucleus
226	>circRNA:chr5:41927012,41927248|circbase_id:hsa_circ_0072371|type:cancer|position:Exon,Intron|host_gene:FBXO4	Nucleus	Nucleus	Nucleus	Cytoplasm
227	>circRNA:chr3:121693826,121694832|circbase_id:--- |type:cancer|position:Exon|host_gene:GOLGB1	Cytoplasm	Cytoplasm	Cytoplasm	Cytoplasm
228	>circRNA:chrX:77988241,78009263|circbase_id:--- |type:cancer|position:Exon,Intron|host_gene:ATP7A	Nucleus	Cytoplasm	Cytoplasm	Cytoplasm
229	>circRNA:chr2:19993067,19997162|circbase_id:--- |type:cancer|position:Exon,Intron|host_gene:MATN3	Cytoplasm	Cytoplasm	Cytoplasm	Cytoplasm
230	>circRNA:chr2:241312438,241312534|circbase_id:--- |type:cancer|position:Intron|host_gene:HDLBP	Cytoplasm	Nucleus	Nucleus	Nucleus
231	>circRNA:chr11:45990792,45990923|circbase_id:--- |type:cancer|position:Intron|host_gene:PHF21A	Nucleus	Nucleus	Nucleus	Nucleus
232	>circRNA:chr16:4798070,4798184|circbase_id:hsa_circ_0037749|type:normal,cancer|position:Exon,Intron|host_gene:AC020663.4	Cytoplasm	Nucleus	Cytoplasm	Nucleus
233	>circRNA:chr14:75030542,75049718|circbase_id:hsa_circ_0032637|type:normal,cancer|position:Exon,Intron|host_gene:MLH3	Nucleus	Cytoplasm	Cytoplasm	Cytoplasm
234	>circRNA:chr9:105570750,105575137|circbase_id:--- |type:normal,cancer|position:Exon,Intron|host_gene:FKTN	Cytoplasm	Cytoplasm	Cytoplasm	Cytoplasm
235	>circRNA:chr10:37856295,37856401|circbase_id:--- |type:normal,cancer|position:Exon,Intron|host_gene:ZNF248	Cytoplasm	Nucleus	Nucleus	Nucleus
236	>circRNA:chr3:139557889,139557965|circbase_id:--- |type:cancer|position:Intron|host_gene:COPB2-DT	Nucleus	Nucleus	Nucleus	Nucleus
237	>circRNA:chr15:72020917,72046634|circbase_id:hsa_circ_0104466|type:	Cytoplasm	Cytoplasm	Cytoplasm	Nucleus

	normal,cancer |position:Exon,Intron|host_gene:MYO9A				
238	>circRNA:chr2:55640626,55673079|circbase_id:---|type:cancer|position:Exon,Intron|host_gene:PNPT1	Nucleus	Cytoplasm	Cytoplasm	Cytoplasm
239	>circRNA:chr17:47328268,47345098|circbase_id:hsa_circ_0044241|type:normal,cancer |position:Exon,Intron|host_gene:EFCAB13	Nucleus	Cytoplasm	Cytoplasm	Cytoplasm
240	>circRNA:chr11:57704718,57704815|circbase_id:---|type:cancer|position:Exon|host_gene:MED19	Cytoplasm	Nucleus	Nucleus	Nucleus
241	>circRNA:chr15:42563748,42563857|circbase_id:---|type:normal,cancer|position:Exon,Intron|host_gene:HAUS2	Nucleus	Nucleus	Nucleus	Cytoplasm
242	>circRNA:chr8:125073152,125084022|circbase_id:hsa_circ_0135706|type:normal,cancer|position:Exon,Intron|host_gene:WASHC5	Cytoplasm	Cytoplasm	Cytoplasm	Cytoplasm
243	>circRNA:chr16:47311239,47313836|circbase_id:hsa_circ_0105438|type:normal,cancer |position:Exon,Intron|host_gene:ITFG1	Nucleus	Cytoplasm	Cytoplasm	Cytoplasm
244	>circRNA:chr15:57708713,57712188|circbase_id:---|type:normal,cancer|position:Exon,Intron|host_gene:POLR2M	Cytoplasm	Cytoplasm	Cytoplasm	Cytoplasm
245	>circRNA:chr4:128884407,129003876|circbase_id:---|type:normal,cancer|position:Exon,Intron|host_gene:SCLT1	Cytoplasm	Cytoplasm	Cytoplasm	Nucleus
246	>circRNA:chr6:43795850,43796560|circbase_id:---|type:cancer|position:Intergenic|host_gene:---	Nucleus	Nucleus	Nucleus	Nucleus
247	>circRNA:chr5:179615545,179621035|circbase_id:---|type:normal,cancer|position:Exon,Intron|host_gene:HNRNPH1	Nucleus	Cytoplasm	Cytoplasm	Cytoplasm
248	>circRNA:chr11:112045137,112053615|circbase_id:---|type:normal,cancer|position:Exon,Intron|host_gene:DLAT	Nucleus	Cytoplasm	Cytoplasm	Cytoplasm
249	>circRNA:chr12:46363244,46363725|circbase_id:---|type:normal,cancer|position:Intron|host_gene:SLC38A2	Nucleus	Nucleus	Nucleus	Nucleus
250	>circRNA:chr13:49114654,49178654|circbase_id:---|type:normal,cancer|position:Exon,Intron|host_gene:FNDC3A	Cytoplasm	Cytoplasm	Cytoplasm	Cytoplasm
251	>circRNA:chr2:158971568,158971703|circbase_id:---|type:normal,cancer|position:Intron|host_gene:TANC1	Nucleus	Nucleus	Nucleus	Nucleus
252	>circRNA:chr15:42449770,42451954|circbase_id:hsa_circ_0034819|type:normal,cancer|position:Exon,Intron|host_gene:ZNF106	Cytoplasm	Cytoplasm	Cytoplasm	Cytoplasm
253	>circRNA:chr20:32795408,32799328|circbase_id:hsa_circ_0059806|type:normal,cancer |position:Exon,Intron|host_gene:DNMT3B	Cytoplasm	Cytoplasm	Cytoplasm	Cytoplasm
254	>circRNA:chr5:179538993,179540832|circbase_id:hsa_circ_0128709|type:normal,cancer|position:Intergenic|host_gene:---	Cytoplasm	Nucleus	Cytoplasm	Cytoplasm
255	>circRNA:chr9:17273732,17487066|circbase_id:---|type:cancer|position:Exon,Intron|host_gene:CNTLN	Nucleus	Cytoplasm	Cytoplasm	Cytoplasm
256	>circRNA:chr4:154542844,154542949|circbase_id:---|type:cancer|position:Intron|host_gene:PLRG1	Nucleus	Nucleus	Nucleus	Nucleus
257	>circRNA:chr2:161176755,161177010|circbase_id:---|type:cancer|position:Intron|host_gene:TANK	Nucleus	Nucleus	Nucleus	Nucleus
258	>circRNA:chr16:21458415,21458520|circbase_id:---|type:cancer|position:Intron|host_gene:SMG1P3	Nucleus	Nucleus	Nucleus	Nucleus
259	>circRNA:chr12:198796,202880|circbase_id:hsa_circ_0098333|type:normal,cancer|position:Exon,Intron|host_gene:SLC6A12	Cytoplasm	Cytoplasm	Cytoplasm	Cytoplasm
260	>circRNA:chr6:166972738,166976385|circbase_id:---|type:normal,cancer|position:Exon,Intron|host_gene:Z94721.1	Nucleus	Nucleus	Cytoplasm	Cytoplasm
261	>circRNA:chr1:20600814,20601012|circbase_id:---|type:cancer|position:Intron|host_gene:CDA	Nucleus	Nucleus	Nucleus	Nucleus
262	>circRNA:chr2:111847137,111850910|circbase_id:--- |type:normal,cancer |position:Exon,Intron|host_gene:ANAPC1	Nucleus	Nucleus	Cytoplasm	Cytoplasm
263	>circRNA:chr1:44739873,44747485|circbase_id:---|type:normal,cancer|position:Exon,Intron|host_gene:KIF2C	Cytoplasm	Cytoplasm	Cytoplasm	Cytoplasm
264	>circRNA:chr17:17223921,17232901|circbase_id:hsa_circ_0042245|type:normal,cancer|position:Exon,Intron|host_gene:AC055811.2	Nucleus	Cytoplasm	Cytoplasm	Cytoplasm
265	>circRNA:chr15:43376502,43386330|circbase_id:---|type:normal,cancer|position:Exon,Intron|host_gene:TUBGCP4	Nucleus	Cytoplasm	Cytoplasm	Cytoplasm
266	>circRNA:chr4:13602689,13603794|circbase_id:---|type:cancer|position:Exon|host_gene:BOD1L1	Nucleus	Cytoplasm	Cytoplasm	Cytoplasm
267	>circRNA:chr6:167832425,167832599|circbase_id:---|type:normal,cancer|position:Intron|host_gene:AFDN	Nucleus	Nucleus	Nucleus	Nucleus
268	>circRNA:chr15:75407054,75430408|circbase_id:hsa_circ_0036349|type:normal,cancer|position:Exon,Intron|host_gene:SIN3A	Cytoplasm	Cytoplasm	Cytoplasm	Cytoplasm
269	>circRNA:chr7:122621480,122624676|circbase_id:---|type:cancer|position:Exon,Intron|host_gene:CADPS2	Nucleus	Cytoplasm	Cytoplasm	Cytoplasm
270	>circRNA:chr12:121714899,121715411|circbase_id:---|type:cancer|position:Intron|host_gene:TMEM120B	Nucleus	Nucleus	Nucleus	Nucleus
271	>circRNA:chr11:3719411,3725219|circbase_id:---|type:normal,cancer|position:Exon,Intron|host_gene:NUP98	Cytoplasm	Cytoplasm	Cytoplasm	Cytoplasm
272	>circRNA:chr3:23370763,23370846|circbase_id:---|type:cancer|position:Intron|host_gene:UBE2E2	Nucleus	Nucleus	Nucleus	Nucleus
273	>circRNA:chr1:18871438,18872413|circbase_id:---|type:cancer|position:Exon|host_gene:ALDH4A1	Nucleus	Nucleus	Cytoplasm	Cytoplasm
274	>circRNA:chr20:58691723,58707200|circbase_id:---|type:cancer|position:Exon,Intron|host_gene:STX16-NPEPL1	Cytoplasm	Cytoplasm	Cytoplasm	Cytoplasm
275	>circRNA:chr20:45894649,45895133|circbase_id:---|type:normal,cancer|position:Exon,Intron|host_gene:CTSA	Cytoplasm	Nucleus	Cytoplasm	Cytoplasm
276	>circRNA:chr16:67144376,67144806|circbase_id:---|type:cancer|position:Intron|host_gene:PHAF1	Nucleus	Nucleus	Nucleus	Cytoplasm
277	>circRNA:chr11:72102924,72103020|circbase_id:---|type:normal,cancer|position:Intron|host_gene:LRTOMT	Cytoplasm	Nucleus	Nucleus	Cytoplasm
